# Differential ubiquitin modifications of ESCRT‐I subunits VPS37‐1 and VPS28‐2 positively regulate plant immunity

**DOI:** 10.1111/nph.71590

**Published:** 2026-09-29

**Authors:** Chaofeng Wang, Yi Zhang, Bangjun Zhou, Pooja Verma, Lirong Zeng

**Affiliations:** ^1^ Department of Plant Pathology, Center for Plant Science Innovation University of Nebraska Lincoln NE 68588 USA

**Keywords:** ESCRT‐I complex, FLS2, PAMP‐triggered immunity, *Pseudomonas syringae* pv *tomato*, VPS28‐2, VPS37‐1, XBAT35.2 ubiquitin ligase

## Abstract

- Sensing of pathogen‐associated molecular patterns (PAMPs) by pattern recognition receptors at the cell surface initiates the first layer of host immunity against invading microbial pathogens. In plants, the immune receptor Flagellin Sensing 2 (FLS2) perceives bacterial flagellin to trigger downstream signaling that culminates in PAMP‐triggered immunity (PTI).
- FLS2 protein levels are critical for effective PTI, yet the regulatory mechanisms governing its stability remain to be fully elucidated. Our previous work showed that the Arabidopsis Really Interesting Gene (RING) type ubiquitin ligase XBAT35.2 positively regulates plant immunity against bacterial pathogen *Pseudomonas syringae* pv *tomato* by promoting FLS2 stability.
- In this study, we demonstrate that XBAT35.2 interacts *in vivo* with two key ESCRT‐I complex components, VPS37‐1 and VPS28‐2. XBAT35.2 ubiquitinates VPS37‐1 with K48‐linked polyubiquitin chains, leading to its proteasomal degradation, and modifies VPS28‐2 with K63‐linked chains, reducing its interaction with FLS2. VPS37‐1 and VPS28‐2 play redundant, negative roles in FLS2‐mediated immunity by promoting vacuolar degradation of FLS2, displaying differential effects on distinct FLS2‐mediated immune responses. By targeting VPS37‐1 and VPS28‐2, XBAT35.2 intercepts this degradative pathway, thereby stabilizing FLS2 and enhancing host immunity.
- These findings uncover a novel regulatory circuit modulating FLS2 abundance and deepen our understanding of controlling the cell‐surface receptor homeostasis in plant immunity.

## Graphical abstract

The RING type ubiquitin ligase (E3), XBAT35.2, positively regulates FLS2‐mediated plant immunity against bacterial pathogens by modifying the ESCRT‐I subunits VPS28‐2 and VPS37‐1 with distinct ubiquitin chain types.

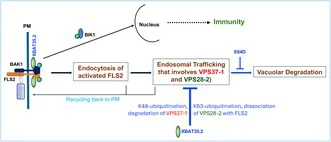

## Introduction

Cell‐surface‐resident pattern recognition receptors (PRRs) are key components of the innate immune system, responsible for detecting conserved microbial features known as pathogen‐associated molecular patterns (PAMPs), such as bacterial flagellin or fungal chitin (DeFalco & Zipfel, 2021; Kawai *et al*., 2024). This recognition initiates PAMP‐triggered immunity (PTI), a defense response that activates a wide array of immune reactions in plants and animals (Ngou *et al*., 2022; Pradeu *et al*., 2024). In plants, one of the best‐characterized PRRs is Flagellin Sensing 2 (FLS2), a leucine‐rich repeat receptor‐like kinase (LRR‐RLK) located at the plasma membrane (PM) that play a critical role in host immunity against bacterial infection (Zipfel *et al*., 2004; Rosli *et al*., 2013). FLS2 specifically binds to flg22, a 22‐amino acid epitope derived from bacterial flagellin, triggering immune activation. FLS2 constitutively interacts with Botrytis‐Induced Kinase 1 (BIK1), a receptor‐like cytoplasmic kinase (RLCK). Upon perception of flg22, FLS2 rapidly forms a complex with the co‐receptor BRI1‐Associated Kinase 1 (BAK1), another PM‐localized LRR‐RLK that also constitutively interacts with BIK1. This FLS2‐BAK1‐BIK1 complex serves as a heterotrimeric signaling hub. Within this complex, sequential autophosphorylation, transphosphorylation, and mono‐ubiquitination events occur – particularly on BIK1 – leading to its dissociation and translocation to the nucleus and the activation of downstream signaling cascades (DeFalco & Zipfel, 2021; Kong *et al*., 2021). These signaling events ultimately culminate in robust host immune responses.

While rapid activation of immune signaling is essential for defense, it must be tightly regulated to prevent excessive or prolonged immune responses that could lead to self‐damage, growth defects, or metabolic burden (Dominguez‐Ferreras *et al*., 2015; Cao, 2016). Thus, plants have evolved sophisticated mechanisms to fine‐tune PRR abundance and activity at the PM, ensuring immune balance is maintained. One of such mechanisms involves endocytosis and subsequent endosomal trafficking of PRRs. For example, both inactive and flg22‐activated FLS2 undergo internalization via clathrin‐mediated endocytosis – a process involving factors like dynamin‐related proteins DRP2B and DRP1A, which have been shown to regulate FLS2 internalization and are essential for flg22‐triggered immune responses (Smith *et al*., 2014; Paez Valencia *et al*., 2016; Ekanayake *et al*., 2021). Internalized FLS2 is then transported through endosomal compartments, including the *trans*‐Golgi network/early endosome (TGN/EE) and late endosomes/multivesicular bodies (LE/MVB) where it is either recycled back from TGN/EE to the PM or targeted for vacuolar degradation (Robatzek *et al*., 2006; Beck *et al*., 2012; Choi *et al*., 2013; Paez Valencia *et al*., 2016). This pathway not only helps maintain PRR homeostasis but also contributes to signaling attenuation. Key components of the endosomal sorting complex required for transport (ESCRT), such as Vacuolar Protein Sorting‐Associated Protein 28‐2 (VPS28‐2; AT4G05000) and VPS37‐1 (AT3G53120), have also been implicated in directing activated FLS2 to MVBs for degradation (Spallek *et al*., 2013). During this process, a large proportion of flg22‐activated FLS2 remains associated with LE/MVBs, further supporting the notion that endosomal trafficking is central to immune regulation.

ESCRT‐mediated cargo sorting is an essential part of the endosomal trafficking system. In line with the expanding roles of endocytosis and endosomal trafficking, the functions of ESCRT – a highly conserved multicomplex machinery – have far‐reaching consequences in intracellular traffic, membrane repair, cell signaling, metabolic regulation, cell division, and genome maintenance (Hurley *et al*., 2025). The ESCRT‐I complex, composed of core subunits, including Tumor Susceptibility Gene 101 (TSG101, also called VPS23), VPS28, VPS37 isoforms (such as VPS37A in mammals), and accessory proteins like MVB12, serves as a master regulator of cellular membrane remodeling, ubiquitin‐dependent cargo sorting, and the biogenesis of intraluminal vesicles in multivesicular endosomes (Bache *et al*., 2004; Wollert & Hurley, 2010; Schmidt & Teis, 2012). Its critical importance in host immunity is highlighted by the fact that while many enveloped viruses hijack ESCRT‐I for their budding and egress, the host repurposes it for precise immune control (Votteler & Sundquist, 2013; Wang *et al*., 2023). In adaptive immunity, ESCRT‐I components like TSG101 mediate the sorting of ubiquitinated T‐cell receptors into micro‐vesicles at the immunological synapse, enabling rapid signal termination to prevent prolonged activation, exhaustion, or aberrant responses (Choudhuri *et al*., 2014). Moreover, VPS37A together with Hepatocyte Growth Factor‐Regulated Tyrosine Kinase Substrate (HGS) and Ubiquitin‐Associated Protein1 (UBAP1) target the key sensor Stimulator of Interferon Genes (STING) for degradation, shutting down type I interferon signaling to curb excessive inflammation and protect against autoimmunity (Gentili *et al*., 2023). In plants, the ESCRT machinery also plays important roles in a variety of cellular and physiological processes, including critical functions in host immunity (Robatzek, 2014; Gao *et al*., 2017).

Despite the importance of the ESCRT machinery in numerous cellular pathways, how its components are post‐translationally modified in these contexts remains a significant knowledge gap. Limited reports indicate that phosphorylation of ESCRT‐III core and accessory components, as well as the plant‐specific, putative ESCRT‐I‐associated component FYVE domain protein required for endosomal sorting 1 (FREE1, also known as FYVE domain‐containing protein 1/FYVE1), is critical for their functions in pathways, such as cytokinetic abscission and abscisic acid (ABA) signaling, respectively (Sun *et al*., 2016; Li *et al*., 2019; Zeng *et al*., 2023). While it is well established that mono‐ and Lys63‐linked ubiquitination of PM proteins serves as a key signal for endocytosis and subsequent sorting of the targeted proteins (Sorkin & von Zastrow, 2009; Paez Valencia *et al*., 2016), modifications of the ESCRT machinery itself by ubiquitin – including ubiquitination of ESCRT‐I components – for regulation of its functions in various pathways remain largely unknown.

Ubiquitination – a highly versatile post‐translational modification – plays a central role in regulating plant immunity. This process involves the sequential action of E1 (activating), E2 (conjugating), and E3 (ligating) enzymes that attach ubiquitin molecules to lysine residues of target proteins through one of the seven lysine residues (K6, K11, K27, K29, K33, K48, and K63) within each ubiquitin molecule to assemble various types of polyubiquitin chains. Canonically, ubiquitination is referred to as formation of K48‐linked polyubiquitin chain, which is the most abundant type of polyubiquitination and is recognized as a principal signal for degradation of targeted proteins via the 26S proteasome. Nonetheless, the types of ubiquitination that occur in a cell are highly diverse and the importance of noncanonical ubiquitination in roles other than proteasome‐dependent protein turnover has been increasingly appreciated (Zhou & Zeng, 2017). It has long been shown that the ubiquitin system is essential for endocytosis of activated FLS2 (Robatzek *et al*., 2006). However, it remains unclear whether the involvement of ubiquitination in FLS2 endocytosis happens at the initial internalization step or the ensuing cargo trafficking and sorting steps, or both. Neither is known whether noncanonical ubiquitination, especially K63‐linked ubiquitination, plays a role in modulating the trafficking and sorting machinery that is critical for endocytosis of various cell‐surface receptors.

The Ankyrin repeats (ANK)‐Really Interesting Gene (RING) type ubiquitin E3 ligase XBAT35 has been demonstrated to modulate plant growth and responses to abiotic and biotic stresses, with functional divergence between its two isoforms, XBAT35.1 and XBAT35.2 that differ only in the presence of a nuclear localization signal (NLS) in the former (Carvalho *et al*., 2012; Yu *et al*., 2016; Liu *et al*., 2017). We previously showed that XBAT35.2, rather than XBAT35.1, promotes plant defense against the bacterial pathogen *Pseudmonas syringae* pv *tomato* (*Pst*) by enhancing accumulation of the FLS2 protein (Zhang *et al*., 2026). However, the corresponding molecular mechanism underpinning this role of XBAT35.2 in plant immunity remains to be fully elucidated. To address this question, we initiated the current study by conducting an immunoprecipitation (IP)‐liquid chromatography‐tandem mass spectrometry (LC‐MS/MS) assay to identify potential XBAT35.2 interactors. Interestingly, three major components of the ESCRT‐I complex – VPS23A, VPS28‐2, and VPS37‐1 – were identified in the assay. VPS37‐1 and VPS28‐2 were previously implicated in the endocytic trafficking of FLS2 (Spallek *et al*., 2013), prompting us to focus on them in this study. Subsequent investigations confirmed the interaction of XBAT35.2 with these two ESCRT‐I components *in planta* and revealed that XBAT35.2 catalyzed K48‐ and K63‐linked ubiquitination of VPS37‐1 and VPS28‐2, respectively, which led to proteasomal degradation of VPS37‐1 and reduced interaction of VPS28‐2 with FLS2. Our results also indicated that VPS37‐1 and VPS28‐2 play redundant, negative roles in FLS2‐mediated immunity by vacuolar breakdown of FLS2. Thus, by sabotaging VPS28‐2/VPS37‐1‐mediated vacuolar degradation of FLS2, XBAT35.2 stabilizes FLS2 to effectively mount immunity against the bacterial pathogen *Pst* DC3000. Given the overarching role of endocytosis and endosomal trafficking/sorting in controlling the protein levels of cell‐surface receptors, our findings support the notion that modification of components of the ESCRT machinery by ubiquitin could be a widespread regulatory mechanism for cell‐surface receptor‐mediated cellular signaling.

## Materials and Methods

### Plant materials

The *Arabidopsis thaliana* T‐DNA insertion mutants *xbat35* (SALK_104813), *vps28‐2* (SALK_040274c), and *vps37‐1.1* (CS804682) were ordered from the Arabidopsis Biological Resource Center (ABRC) at Ohio State University and genotyped with T‐DNA and gene‐specific primers (Supporting Information Table S1). The double mutants *vps28‐2*/*vps37‐1.1* were generated by crossing corresponding homozygous single mutants. The *35S:VPS28‐2‐HA* and *35S:VPS37‐1‐HA*, respectively, were introduced into Columbia‐0 (Col‐0), *xbat35*, and *XBAT35.2‐FLAG*‐overexpressing plants to generate corresponding *VPS28‐2* and *VPS37‐1*‐overexpression lines. *Arabidopsis* seeds were surface‐sterilized with 10% (v/v) bleach including 0.2% (v/v) Triton‐100 for 8 min, washed thoroughly in sterile water for 4–6 times before sown in soil or germinated on ½‐strength Murashige & Skoog (½MS) medium (pH 5.8–6.0), and grown in the reach‐in growth chamber at 22°C and *c*. 65% relative humidity under a 16 h : 8 h, day : night cycle. *Nicotiana benthamiana* plants were grown in soil in a walk‐in growth chamber at 26°C under a 16 h : 8 h, day : night long‐day cycle.

### DNA manipulations and plasmid constructions

All DNA manipulations were performed according to standard techniques (Green *et al*., 2012). The constructs for bimolecular fluorescence complementation (BiFC) assay were prepared by PCR amplification of cDNA and cloning the coding sequence of the corresponding genes into the pSPYCE(M) and pSPYNEC 173 vectors (Waadt *et al*., 2008). To generate constructs for expression and purification of recombinant proteins, coding sequences of the corresponding genes were amplified from cDNA and cloned into the pDEST15 (Thermo Fisher, Waltham, MA, USA), pDEST17 (Thermo Fisher), and pMAL‐c2 (New England Biolabs, Ipswich, MA, USA) to create translational fusion to the GST, 6HIS, and maltose‐binding protein (MBP) epitope tag, respectively. Constructs for overexpressing XBAT35.2‐FLAG in plants were generated by cloning the coding sequences into the pBTEX vector (Frederick *et al*., 1998). Constructs overexpressing VPS28‐2 and VPS37‐1 were generated by PCR amplifying and cloning the corresponding coding sequences into the pBTEX1300HA vector in which a hemagglutinin (HA) epitope tag is included to be fused in frame to the protein.

### Immunoblotting and antibody resource

Immunoblotting was performed according to standard techniques (Green *et al*., 2012) using appropriate antibody. Simply, equal plant samples were used for extraction of total proteins, and the total protein concentration was measured for all samples to ensure equal loading in every experiment. For measuring the relative abundance of the FLS2 protein in immunoblot, the pixel density of the FLS2 band was quantified and normalized to the pixel density of the corresponding Rubisco band using the imagej software (Schneider *et al*., 2012), with the normalized value in untreated Col‐0 plants being set as 1. The anti‐HA (clone 3F10), peroxidase‐conjugated monoclonal antibody was purchased from Roche. The anti‐ubiquitin (MilliporeSigma, Burlington, MA, USA), anti‐MBP (New England Biolabs), anti‐GFP (Santa Cruz Biotechnology, Dallas, TX, USA) antibodies were ordered from corresponding companies. The anti‐FLS2 antibodies were purchased from Agrisera. The goat (*Capra hircus* L.) peroxidase‐conjugated anti‐rabbit (*Lepus Cuniculus* L.) IgG (H + L) secondary antibody and goat peroxidase‐conjugated anti‐mouse (*Mus musculus* L.) IgG (H + L) secondary antibody were purchased from Bio‐Rad.

### Quantitative real‐time PCR

Seven‐day‐old Arabidopsis seedlings grown on ½MS plates were immersed in 1 ml sterilized deionized H_2_O in a 24‐well plate. After overnight incubation at room temperature, H_2_O was changed into 1 μM flg22 solution. Sterilized deionized H_2_O was used as negative control for the flg22 treatment. At the indicated time points, seedlings were sampled and subjected to total RNA extraction using TRIzol Reagent (MilliporeSigma) according to the manufacturer's instruction. First‐strand cDNA synthesis was performed using 2 μg of total RNA, oligo(dT) 18 primers, and the GoScript™ Reverse Transcriptase (Promega). Quantitative reverse transcription polymerase chain reaction (qRT‐PCR) was performed using gene‐specific primers (Table S1) and SYBR Green (Life Technologies, Waltham, MA, USA) on a Bio‐Rad iQ5 PCR machine by following the manufacturer's protocol.

### Pathogen growth assay

The bacterial pathogen *Pseudmonas syringae* pv *tomato* (Okabe) Young, Dye & Wilkie strain DC3000 cells were cultured on King's B (KB) agar plate with rifampicin (50 mg l^−1^) at 28°C for 2 d. The bacterial cells were then collected, resuspended in 10 mM MgCl_2_ containing 0.02% Silwet L‐77, and diluted to the concentration at OD600 of 0.2. Approximately 4‐wk‐old *Arabidopsis* plants with or without 1 μM flg22 treatment for 2 h were sprayed with the pathogen inoculum and kept under high humidity for disease development. For measuring bacterial growth, the leaves of inoculated plants were surface‐sterilized with 70% ethanol for 1 min and rinsed with sterile water for 1 min before three leaf disks were collected using puncher at 2‐ and 3‐d postinfection. The leaf disks were thoroughly ground in a 1.5‐ml Eppendorf tube and serially diluted and plated on KB plates with rifampicin (25 mg l^−1^) for growing of the pathogen at 28°C for *c*. 2 d, followed by counting the number of colonies and data analysis.

### Transient protein expression in *Nicotiana benthamiana*

The constructs were transformed into *Agrobacterium tumefaciens* (Smith & Townsend) Conn strain GV3101 by electroporation. A 5‐ml seed LB culture of the transformed *A. tumefaciens* cells was grown at 28°C overnight with appropriate antibiotics presented. The seed culture was then transferred into a suitable amount of LB medium containing appropriate antibiotics, 10 mM MES, and 20 mM acetosyringone the next day for shaker growing at 28°C for 24 h. The cultured bacteria were then collected by centrifugation at 3800 ***g*** for 15 min. The collected cells were resuspended in infiltration solutions (10 mM MgCl_2_, 10 mM MES, and 200 mM acetosyringone) to appropriate OD600 values and kept at room temperature for at least 2 h, followed by Agro‐infiltration into 4‐wk‐old tobacco leaves using needleless 1‐ml syringes.

### Bimolecular fluorescence complementation assay

For the assay using *N. benthamiana* leaves, N‐terminal yellow fluorescent protein (YFP)‐fused and C‐terminal YFP‐fused proteins were transiently co‐expressed in the leaves of 4‐wk‐old *N. benthamiana* plants by Agro‐infiltration. N‐terminal YFP‐fused and C‐terminal β‐glucuronidase (GUS) proteins or empty vectors were used as negative controls. The images were taken 48 h after infiltration. Confocal imaging was carried out using the Nikon A1‐NiE upright fluorescence microscope confocal system with the following excitation and emission wavelengths: YFP, 514.5 nm (excitation) and 525 to 555 nm (emission); Chl autofluorescence, 640.5 nm (excitation), and 663 to 738 nm (emission).

### Detection of ROS production by luminol‐based assay

Reactive oxygen species (ROS) production was measured using a luminol‐based chemiluminescence assay according to Gómez‐Gómez *et al*. (1999), with minor modifications. A single 1/4‐inch‐diameter leaf disk was excised from fully expanded leaves of 4‐wk‐old Arabidopsis plants and placed in a well of a 96‐well tissue culture plate containing 1 ml of sterile water. After *c*. 16 h overnight adaptation, the water was removed, and 1 ml of assay solution containing 10 μM luminol, 10 μg ml^−1^ horseradish peroxidase (HRP), and 1 μM flg22 was added to each well. Luminescence was immediately recorded for 30 min using a photon‐counting camera (Photek, East Sussex, UK).

### Co‐immunoprecipitation assay and LC‐MS/MS

For co‐immunoprecipitation (Co‐IP) using tobacco leaves, different combinations of *A. tumefaciens* carrying the constructs of interest were co‐infiltrated into tobacco leaves. Green fluorescent protein (GFP) was used as internal expression control. Total proteins were then extracted 2 d after infiltration using Co‐IP extraction buffer containing 50 mM Tris–HCl, pH 7.5, 150 mM NaCl, 10% glycerol, 10 mM DTT, 1 mM NaF, 1 mM Na_2_MoO_4_.2H_2_O, 1% (v/v) Phosphatase Inhibitor Cocktails (Sigma‐Aldrich), 1% (v/v) Protease Inhibitor Cocktail (P9599; Sigma‐Aldrich), 100 μM phenylmethylsulphonyl fluoride and 0.5% (v/v) Triton‐100. For Co‐IP assays in Arabidopsis, leaves from Col‐0 and 35S:*XBAT35.2* transgenic plants were treated with flg22 for 1 h, after which total proteins were extracted using Co‐IP extraction buffer. Supernatants were incubated for 3–4 h at 4°C with 15–20 μl of ANTI‐FLAG® M2 Affinity Gel (Sigma) and washed three to four times with Co‐IP wash buffer containing 0.1% Triton‐100 but without Phosphatase Inhibitor Cocktails and Protease Inhibitor Cocktail. The immunoprecipitated protein sample was incubated in 1×SDS loading buffer at 95°C for 5 min and centrifuged for 1 min at 14 000 ***g***, and then, the supernatants were analyzed by immunoblotting with an anti‐HA or anti‐FLAG antibody.

MS/MS samples were analyzed using Mascot (v.2.7.0; Matrix Science, London, UK). Mascot was set up to search the predicted trypsin‐digested *N. benthamiana* proteome database (Wang *et al*., 2024), with a fragment ion mass tolerance of 20 PPM and a parent ion tolerance of 20 PPM. Scaffold (v.Scaffold_5.2.2, Proteome Software Inc., Portland, OR, USA) was used to validate MS/MS‐based peptide and protein identifications. Peptide identifications were accepted if they could be established at > 80.0% probability by the Peptide Prophet algorithm (Keller *et al*., 2002) with Scaffold delta‐mass correction. Protein identifications were accepted if they could be established at greater than 99.0% probability and contained at least two identified peptides. Protein probabilities were assigned by the Protein Prophet algorithm (Nesvizhskii *et al*., 2003). Proteins that contained similar peptides and could not be differentiated based on MS/MS analysis alone were grouped to satisfy the principles of parsimony. Proteins sharing significant peptide evidence were grouped into clusters.

### Protein degradation Assay

For examining the possible degradation of VPS37‐1 and VPS28‐2 by XBAT35.2 *in planta*, the experiments were performed as described previously with some modifications (Yu *et al*., 2020). In brief, different combinations of *A. tumefaciens* carrying constructs of interest were co‐infiltrated into tobacco leaves. GFP was used as an internal expression control. Leaf samples were collected 2 d after the infiltration. Total proteins were then extracted from the leaves using the protein extraction buffer employed for the Co‐IP assays and subjected to immunoblotting using anti‐HA, anti‐FLAG, and anti‐GFP antibodies, respectively. Coomassie Brilliant Blue (CBB) staining for the Rubisco proteins was used as a loading control.

### Recombinant protein expression and purification

All GST‐tagged (pDEST15), His‐tagged (pDEST17), and MBP‐tagged (pMAL‐C2) fusion proteins were expressed using the BL21 *Escherichia coli* strain, and the affinity purification was performed using standard protocols. The protein concentration was determined using Bio‐Rad protein assay dye reagent concentrate (Bio‐Rad).

### *In vitro* and *in vivo* ubiquitination assays

The *in vitro* ubiquitination assay was performed as described previously (Mural *et al*., 2013). In brief, the reaction was performed in 30 μl ubiquitination buffer (50 mM Tris–HCl, pH 7.5, 5 mM ATP, 5 mM MgCl_2_, 2 mM DTT, 3 mM Creatine phosphate, and 5 μg ml^−1^ of Creatine phosphokinase) containing 10 μg of ubiquitin, 40 ng of E1, 200 ng of E2, 3 μg of E3, and 200 ng of substrates at 30°C for 3 h. The reactions were stopped by adding 10 μl 4× SDS loading buffer and heating at 95°C for 5 min. Five to ten microliters of reaction sample were used for immunoblotting analysis. For detecting the ubiquitination of VPS28‐2 and VPS37‐1 by XBAT35.2 *in planta*, the seedlings of Arabidopsis transgenic lines where VPS28‐2 or VPS37‐1‐HA expressed in the genetic background of Col‐0, *xbat35*, and *35S:XBAT35.2‐FLAG*, respectively, were grown on ½MS medium plus agar plates for 10 d under long‐day condition, then transferred to a flask with liquid ½MS medium containing 75 μM MG132 and shaken overnight. Total proteins were extracted from the MG132‐treated seedlings using the extraction buffer as described previously with minor modification by adding 75 μM MG132 (Aguilar‐Hernández *et al*., 2017), followed by immunoblotting analysis using anti‐HA antibodies.

### Data statistical analysis

Statistical significance among treatments was determined by one‐way ANOVA (Tukey HSD), and asterisks indicate the statistical significance: *, *P* < 0.05; **, *P* < 0.01.

## Results

### VPS37‐1 and VPS28‐2 are co‐immunoprecipitated and interact with XBAT35.2 *in planta*

Our previous studies showed that the ubiquitin ligase XBAT35.2 promotes accumulation of the immune receptor FLS2, especially when exposed to pathogenic bacteria, without directly ubiquitinating FLS2 *in vitro* (Zhang *et al*., 2026). These observations suggest that XBAT35.2 stabilizes FLS2 indirectly by targeting intermediary or ‘third party’ protein(s) that control FLS2 levels. To identify such proteins and elucidate the molecular basis of XBAT35.2's positive role in FLS2‐mediated immunity, we performed Co‐IP assays followed by LC‐MS/MS to identify XBAT35.2‐associated proteins using both *N. benthamiana* leaves transiently expressing XBAT35.2‐FLAG and an Arabidopsis transgenic line overexpressing XBAT35.2‐FLAG (Zhang *et al*., 2026). To prevent proteasome‐mediated degradation of potential interactors during pull‐down (due to XBAT35.2‐mediated ubiquitination), plants were treated with the proteasome inhibitor MG132 or a mock solution after flg22 elicitation.

In addition to the ubiquitin‐26S proteasome system, endocytosis‐mediated recycling to PM and lysosomal/vacuolar degradation also play important roles in regulating levels of PM‐bound receptors (Paez Valencia *et al*., 2016; von Zastrow & Sorkin, 2021). Therefore, we focused particular attention in our co‐IP‐LC‐MS/MS analyses on components of the endocytosis and endocytic trafficking machinery, including constituents of the ESCRT‐I complex, VPS37‐1 and VPS28‐2, which have been implicated in FLS2‐mediated PTI (Spallek *et al*., 2013). Data mining and sequence analysis of the *N. benthamiana* genome (Wang *et al*., 2024) revealed four VPS28‐2 orthologs (Nbe03g32210, Nbe10g10130, Nbe17g20060, and Nbe18g25060) and three VPS37‐1 orthologs (Nbe10g05990, Nbe13g03380, and Nbe15g09500) that share high sequence homology with their Arabidopsis counterparts. We designated these as VPS28‐2.1‐2.4 and VPS37‐1.1‐1.3, respectively (Datasets S1 and S2). This strong conservation between the Arabidopsis and *N. benthamiana* ESCRT‐I proteins supported the use of tobacco as a reliable heterologous system for identifying potential XBAT35.2 interactors.

LC‐MS/MS analysis revealed that the core ESCRT‐I subunits VPS37‐1 and VPS23A were significantly enriched in *N. benthamiana* XBAT35.2‐FLAG pull‐down samples treated with MG132 (Fig. 1a; Dataset S3A). To confirm the specificity of these interactions, we performed a separate Co‐IP assay in *N. benthamiana* using GFP‐FLAG as a negative control. In the assay, both VPS37‐1 and VPS23A were consistently detected in the XBAT35.2‐FLAG pull‐down samples but were absent in the GFP‐FLAG control samples. Notably, VPS28‐2 was also identified as an interactor in this independent assay (Fig. S1a,b; Dataset S3B). However, the peptide counts for VPS28‐2, VPS37‐1, and VPS23A were slightly lower than in the initial experiment, most likely due to lower expression levels of XBAT35.2‐FLAG in this replicate, resulting in fewer protein baits available for pull‐down (Fig. S1a,b). VPS23A has been shown to regulate the turnover of ABA receptors and is itself a substrate for XBAT35‐mediated degradation (Yu *et al*., 2016). The identification of VPS23A and ACD11 – another protein previously reported to be targeted by XBAT35.2 – further validates the reliability of our co‐IP assay (Table S2). Notably, VPS28‐2 and VPS37‐1 were also enriched in co‐IP pull‐down samples from *XBAT35.2‐FLAG*‐overexpressing *Arabidopsis* lines, further supporting genuine interactions between XBAT35.2 and both VPS37‐1 and VPS28‐2 *in planta* (Fig. S1c; Dataset S4). VPS37‐1 and VPS28‐2 have been implicated in endosomal sorting of flg22‐activated FLS2 (Spallek *et al*., 2013). Based on these findings, we focused our subsequent investigation on the interaction between XBAT35.2 and the ESCRT‐I subunits VPS37‐1 and VPS28‐2.

**Fig. 1:**
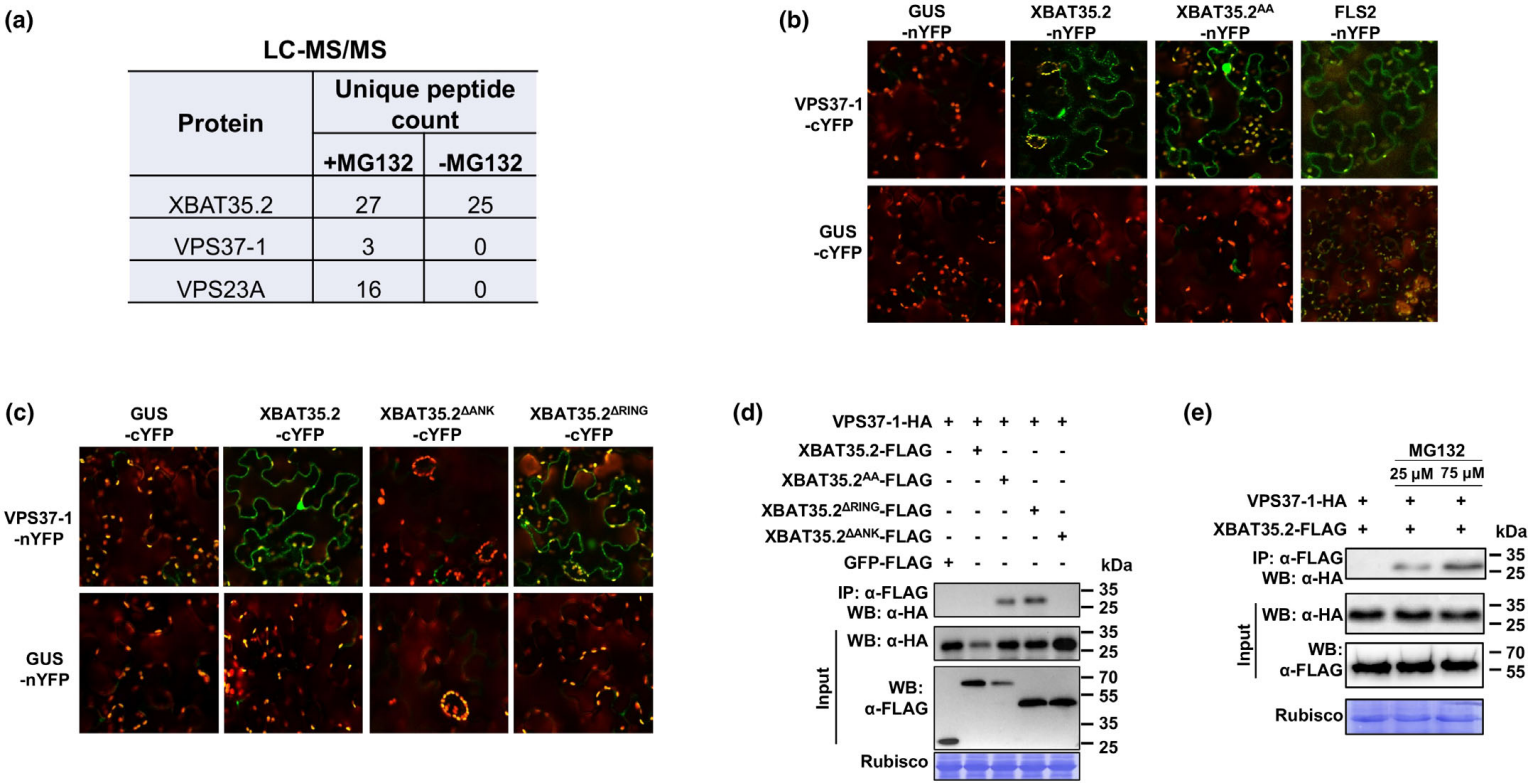
XBAT35.2 specifically interacts with VPS37‐1 *in vivo*. (a) Number of XBAT35.2, VPS37‐1, and VPS23a peptides identified by immunoprecipitation (IP)‐liquid chromatography‐tandem mass spectrometry (IP‐LC‐MS/MS). *Agrobacterium tumefaciens* cells carrying XBAT35.2–FLAG constructs were infiltrated into *Nicotiana benthamiana* leaves. At 48 h post‐infiltration, leaves were treated with 1 μM flg22 for 1 h before sampling. Total proteins were extracted and subjected to IP using an anti‐FLAG antibody, followed by LC‐MS/MS analysis. (b) Bimolecular fluorescence complementation (BiFC) analysis of the interaction of cYFP‐fused VPS37‐1 with nYFP‐fused XBAT35.2, and its E3 ligase activity null mutant (XBAT35.2^AA^) in tobacco leaf epidermal cells. Yellow fluorescent protein (YFP) signal (pseudo‐colored in green) indicates interaction between the two proteins tested. The tobacco leaves were collected and examined on the third day after Agro‐infiltration. β‐glucuronidase (GUS) and Flagellin Sensing 2 (FLS2) were used as negative and positive control, respectively. (c) BiFC analysis of the interaction of nYFP‐fused VPS37‐1 with cYFP‐fused XBAT35.2 and its truncation mutants (XBAT35.2^ΔANK^ and XBAT35.2^ΔRING^) in tobacco leaf epidermal cells. YFP signal (pseudo‐colored in green) indicates interaction between the two proteins tested. The tobacco leaves were collected and examined on the third day after Agro‐infiltration. GUS was used as negative control. (d) Co‐immunoprecipitation (Co‐IP) assay confirms the interaction of XBAT35.2 with VPS37‐1 *in vivo*. *Agrobacterium tumefaciens* cells harboring the constructs that express VPS37‐1‐HA and XBAT35.2‐FLAG, XBAT35.2^ΔANK^‐FLAG, XBAT35.2^ΔRING^‐FLAG, or XBAT35.2^AA^‐FLAG were co‐infiltrated into tobacco leaves. Total protein was extracted from leaves on the third day after Agro‐infiltration. Co‐immunoprecipitation (Co‐IP) was carried out with anti‐FLAG antibody, and the immunoprecipitated samples were analyzed by immunoblotting using anti‐HA antibody. GFP‐FLAG was used as negative control. (e) MG132 treatment promotes the Co‐IP of VPS37‐1 with XBAT35.2. *Agrobacterium tumefaciens* cells harboring the constructs that express VPS37‐1‐HA and XBAT35.2‐FLAG were co‐infiltrated into tobacco leaves. Total protein was extracted from leaves on the third day after Agro‐infiltration. MG132 was added to the protein extraction buffer at indicated concentration. Co‐IP was carried out with anti‐FLAG antibody, and the immunoprecipitated samples were analyzed by immunoblotting using anti‐hemagglutinin (HA) antibody.

We first examined the *in vivo* interaction of XBAT35.2 with VPS37‐1 using the BiFC assay. As shown in Fig. 1(b), confocal microscopy showed reconstituted YFP fluorophore between VPS37‐1‐cYFP and XBAT35.2‐nYFP, implying that the two proteins associate with each other. Strong interactions were detected between VPS37‐1‐cYFP and the E3‐ligase‐inactive mutant XBAT35.2^AA^‐nYFP used in a previous study (Liu *et al*., 2017), suggesting that the interaction of XBAT35.2 with VPS37‐1 is independent of its E3 activity. To further study the protein domains through which XBAT35.2 interacts with VPS37‐1, we employed the truncations of XBAT35.2, XBAT35.2^ΔANK^, and XBAT35.2^ΔRING^ for the BiFC assay (Zhang *et al*., 2026). As shown in Fig. 1(c), VPS37‐1‐nYFP showed interaction with XBAT35.2^ΔRING^‐cYFP, but not with XBAT35.2^ΔANK^‐cYFP, demonstrating that the ANK repeats domain of XBAT35.2 is required for interaction with VPS37‐1 and consistent with the known role of ANKs in mediating protein–protein interactions (Kane & Spratt, 2021). No fluorescence signals were detected when negative control GUS‐cYFP or GUS‐nYFP was co‐expressed with corresponding split YFP‐tagged VPS37‐1 or XBAT35.2 in the assay (Fig. 1b,c).

Besides VPS37‐1, the ESCRT‐I component VPS28‐2 has also been implicated in the endosomal trafficking/sorting of FLS2 and was detected in our co‐IP/LC‐MS/MS assay (Fig. S1b,c; Datasets S3, S4; Spallek *et al*., 2013). We therefore tested the *in vivo* interaction between XBAT35.2 and VPS28‐2 using BiFC. Like VPS37‐1, VPS28‐2 was shown to interact with XBAT35.2 *in vivo* in an ANK domain‐dependent manner (Fig. 2a,b). No YFP fluorescence signals were detected in the negative controls using GUS‐cYFP or GUS‐nYFP (Fig. 2a,b). Consistent with a previous report (Spallek *et al*., 2013), FLS2 (as positive control) interacted with VPS37‐1 and VPS28‐2 in the BiFC assay (Figs 1b, 2a).

**Fig. 2:**
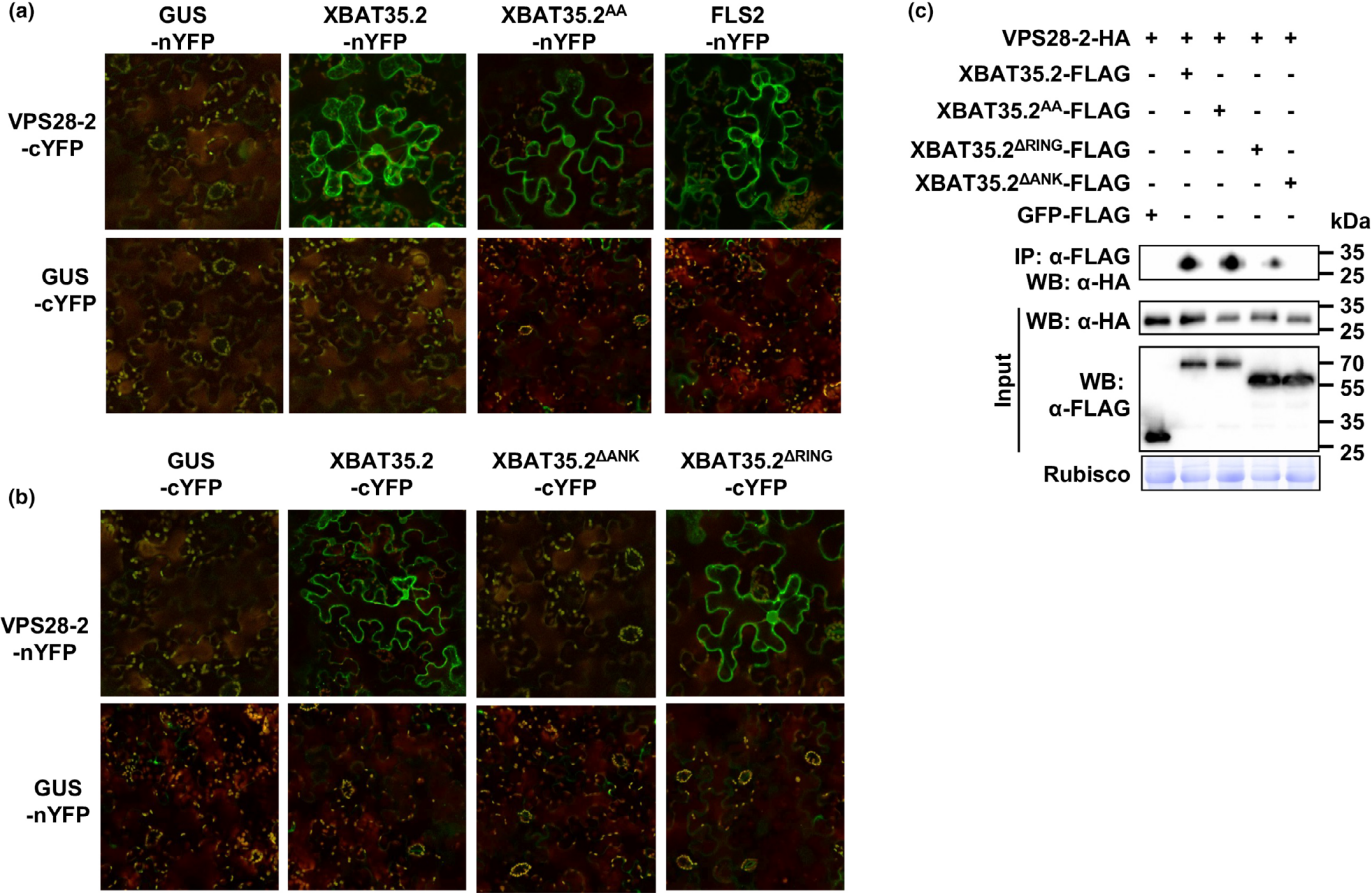
XBAT35.2 specifically interacts with VPS28‐2 *in vivo*. (a) Bimolecular fluorescence complementation (BiFC) analysis of the interaction of cYFP‐fused VPS28‐2 with nYFP‐fused XBAT35.2 and its E3 ligase activity null mutant (XBAT35.2^AA^) in tobacco (*Nicotiana benthamiana*) leaf epidermal cells. Yellow fluorescent protein (YFP) signal (pseudo‐colored in green) indicates interaction between the two proteins tested. The tobacco leaves were collected and examined on the third day after Agro‐infiltration. β‐glucuronidase (GUS) and Flagellin Sensing 2 (FLS2) were used as negative and positive control, respectively. (b) BiFC analysis of the interaction of nYFP‐fused VPS28‐2 with cYFP‐fused XBAT35.2 and its truncation mutants (XBAT35.2^ΔANK^ and XBAT35.2^ΔRING^) in tobacco leaf epidermal cells. YFP signal (pseudo‐colored in green) indicates interaction between the two proteins tested. The tobacco leaves were collected and examined on the third day after Agro‐infiltration. GUS was used as a negative control. (c) Co‐immunoprecipitation (Co‐IP) assay confirms the interaction of XBAT35.2 with VPS28‐2 *in vivo*. *Agrobacterium tumefaciens* cells harboring the constructs that express VPS28‐2‐HA and XBAT35.2‐FLAG, XBAT35.2^ΔANK^‐FLAG, XBAT35.2^ΔRING^‐FLAG, or XBAT35.2^AA^‐FLAG were co‐infiltrated into tobacco leaves. Total protein was extracted from leaves on the third day after Agro‐infiltration. Co‐immunoprecipitation was carried out with anti‐FLAG antibody, and the immunoprecipitated samples were analyzed by immunoblotting using anti‐hemagglutinin (HA) antibody. GFP‐FLAG was used as a negative control.

To further assess the reliability and specificity of the XBAT35.2 association with VPS37‐1 and VPS28‐2, we performed BiFC assays to test for possible interactions of XBAT35.2 with DRP1A and DRP2B – two dynamin‐related proteins required for FLS2 endocytosis and FLS2‐mediated immunity (Smith *et al*., 2014; Ekanayake *et al*., 2021). No YFP signals were observed when DRP1A‐cYFP or DRP2B‐cYFP was co‐expressed with XBAT35.2‐nYFP in the assay (Fig. S2). By contrast, strong YFP signals were detected when DRP1A‐cYFP or DRP2B‐cYFP was co‐expressed with FLS2‐nYFP, consistent with prior findings (Smith *et al*., 2014; Ekanayake *et al*., 2021).

The interactions of XBAT35.2 with VPS37‐1 and VPS28‐2 were further confirmed by Co‐IP assays. As shown in Fig. 1d, VPS37‐1‐HA could be co‐immunoprecipitated by XBAT35.2^AA^‐FLAG and XBAT35.2^ΔRING^‐FLAG but not by XBAT35.2^ΔANK^‐FLAG, corroborating the BiFC assay results (Fig. 1c) that the ANK domain of XBAT35.2 is required for interaction with VPS37‐1. Notably, VPS37‐1‐HA was not co‐immunoprecipitated by wild‐type (WT) XBAT35.2‐FLAG (Fig. 1d). However, treatment with MG132 restored co‐precipitation of VPS37‐1 with XBAT35.2 (Fig. 1e). These results suggest that VPS37‐1 is likely a direct ubiquitination target of XBAT35.2 and that, in the absence of proteasome inhibition, the association of enriched XBAT35.2 with VPS37‐1 during Co‐IP led to the ubiquitination and degradation of VPS37‐1. Co‐IP of VPS28‐2‐HA with XBAT35.2‐FLAG, XBAT35.2^AA^‐FLAG, and XBAT35.2^ΔRING^‐FLAG was also readily detected (Fig. 2c), consistent with the BiFC assay results (Fig. 2a,b). The stable Co‐IP of XBAT35.2 with VPS28‐2 implies that VPS28‐2 is unlikely to be targeted by XBAT35.2 for degradation *in planta*.

Our previous study demonstrated that XBAT35.2 functions in FLS2‐mediated plant immunity against the bacterial pathogen *Pseudomonas syringae* pv *tomato* DC3000 (*Pst* DC3000; Zhang *et al*., 2026). We therefore examined whether activation of plant immunity by *Pst* DC3000 influences the interactions of XBAT35.2 with VPS28‐2 and VPS37‐1. Co‐IP assays revealed that *Pst* DC3000 inoculation enhanced the interaction of VPS28‐2 with both XBAT35.2‐FLAG and XBAT35.2^AA^‐FLAG (Fig. S3). By contrast, *Pst* DC3000 enhanced the interaction of VPS37‐1 only with XBAT35.2^AA^‐FLAG, whereas its interaction with XBAT35.2‐FLAG appeared to be modestly reduced compared with mock‐treated samples. These results are consistent with those shown in Fig. 1(d,e) and further support the hypothesis that VPS37‐1 is targeted for degradation by XBAT35.2 *in planta*. They also suggest that VPS28‐2 and VPS37‐1 function together with XBAT35.2 to regulate FLS2‐mediated immunity against *Pst* DC3000.

### XBAT35.2 modifies VPS37‐1 and VPS28‐2 with distinct ubiquitin chain types

We next examined whether XBAT35.2 can ubiquitinate VPS37‐1 and VPS28‐2 using *in vitro* ubiquitination assays. As shown in the lower panel of Fig. 3(a), in the presence of E1 (GST‐AtUBA1), E2 (GST‐AtUBC12), ubiquitin, and other essential factors, MBP‐VPS37‐1 was conjugated with ubiquitin chains when GST‐XBAT35.2 was included, but not in its absence. By contrast, the MBP tag alone (negative control) showed no ubiquitination by XBAT35.2, even though strong autoubiquitination was detected for XBAT35.2 (Fig. 3a, upper panel). The relatively weak ubiquitination signal for VPS37 by XBAT35.2 was reminiscent of a previous report showing only faint ubiquitination of VPS23A by XBAT35 in *in vitro* ubiquitination assays (Yu *et al*., 2020).

**Fig. 3:**
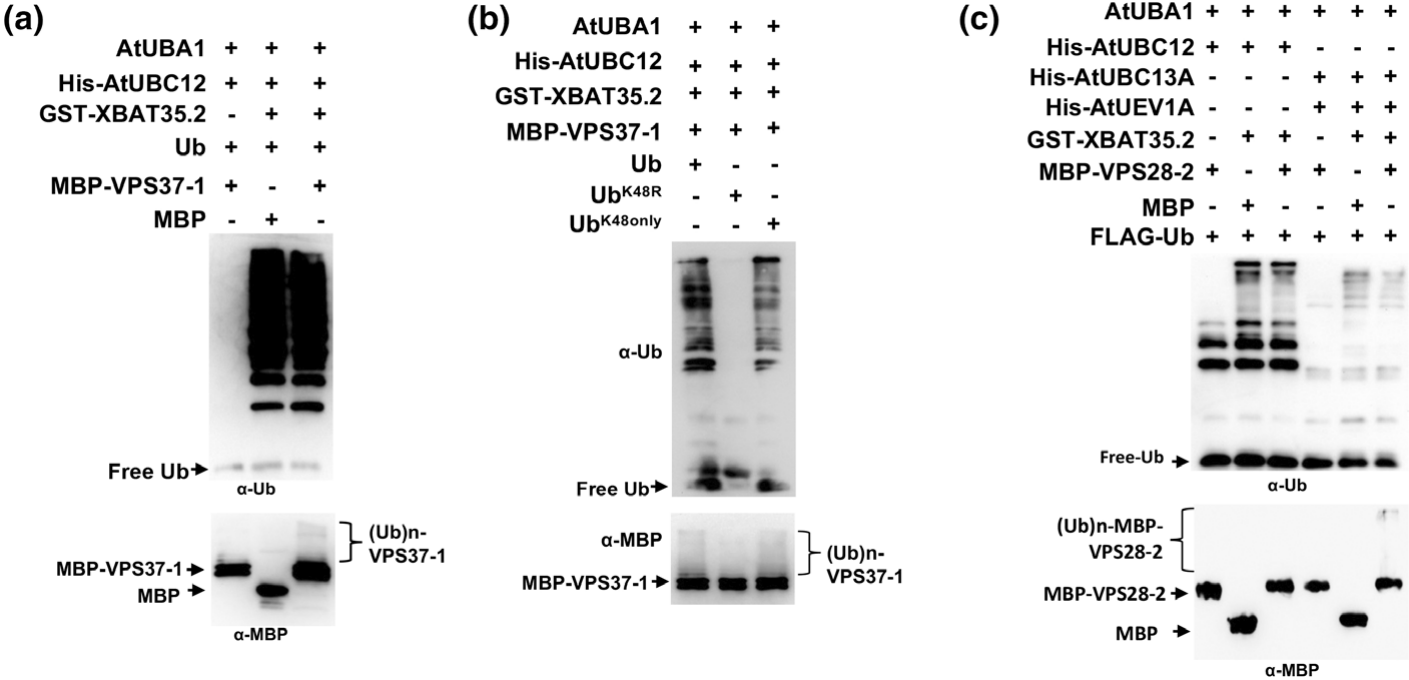
XBAT35.2 directs ubiquitination of VPS37‐1 and VPS28‐2 using distinct ubiquitin chains. (a) Arabidopsis XBAT35.2 ubiquitinates VPS37‐1 *in vitro*. Upper panel: XBAT35.2 autoubiquitination detected by anti‐ubiquitin antibody denotes E3 ligase activity of XBAT35.2. Lower panel: The high molecular weight smear marks ubiquitination of VPS37‐1 ((Ub)n‐VPS37‐1). Maltose‐binding protein (MBP) was used as negative control. (b) XBAT35.2 ubiquitinate VPS37‐1 with K48‐linked ubiquitin chains. Upper panel: XBAT35.2 autoubiquitination detected by anti‐ubiquitin antibody. Lower panel: The high molecular weight smear marks ubiquitination of VPS37‐1 ((Ub)n‐VPS37‐1). MBP was used as negative control. (c) XBAT35.2 ubiquitinate VPS28‐2 *in vitro* through K63‐linked ubiquitin chains. Upper panel: XBAT35.2 autoubiquitination detected by anti‐ubiquitin antibody denotes E3 ligase activity of XBAT35.2. Lower panel: The high molecular weight smear represents ubiquitination of VPS28‐2 ((Ub)n‐VPS28‐2). MBP was used as negative control.

To determine the type of ubiquitin linkage conjugated to VPS37‐1 protein, we used ubiquitin variants Ub^K48R^ (K48 mutated to R) and Ub^K48only^ (all lysine residues except K48 mutated to R). These assays clearly demonstrated that VPS37‐1 can be modified with K48‐linked ubiquitin chains (Figs 3b, S4). In parallel experiments, we used ubiquitin E2 AtUBC13A and its cofactor AtUEV1a (which direct exclusive K63‐linked ubiquitination) and ubiquitin variants Ub^K63R^ and Ub^K63only^ (Fig. S4). While strong XBAT35.2 autoubiquitination was observed in the assay (Lanes 1, 3, 5, and 7), no ubiquitination of VPS37‐1 was detected (Lanes 2 and 4), indicating VPS37‐1 is not modified by XBAT35.2 via K63‐linked ubiquitin chains. Importantly, VPS28‐2 was ubiquitinated by XBAT35.2 by K63‐linked ubiquitin chains when AtUBC13A and its cofactor AtUEV1a were employed (Fig. 3c). By contrast, when the E2 UBC12 (which directs K48‐ but not K63‐linked ubiquitination) was used, no ubiquitination of VPS28‐2 occurred.

To confirm the effects of XBAT35.2 E3 activities on VPS37‐1 and VPS28‐2 *in planta*, we generated stable transgenic Arabidopsis lines expressing *35S:VPS37‐1‐HA* or *35S:VPS28‐2‐HA* constructs in Col‐0, the *xbat35* mutant, and *35S:XBAT35.2‐FLAG* backgrounds. Immunoblotting showed that the ubiquitination of VPS37‐1 and VPS28‐2 was markedly stronger in the *35S:XBAT35.2‐FLAG* plants and significantly reduced in the *xbat35* mutant than that in Col‐0 (Fig. S5). These results indicate that VPS37‐1 and VPS28‐2 are ubiquitinated by XBAT35.2 *in vivo*.

### XBAT35.2‐mediated ubiquitination results in degradation of VPS37‐1 and significantly reduces interaction of VPS28‐2 with FLS2

K48‐linked polyubiquitin chains typically signal 26S proteasome‐dependent protein degradation, whereas K63‐linked ubiquitination generally mediates nonproteolytic regulatory functions, such as modulating protein interactions or localization. To determine whether XBAT35.2 promotes degradation of VPS37‐1 and VPS28‐2, we performed dose‐dependent degradation assays by co‐expressing VPS37‐1‐HA or VPS28‐2‐HA with increasing amounts of XBAT35.2‐FLAG in tobacco leaves. The abundance of VPS37‐1‐HA decreased progressively with increasing XBAT35.2 (Fig. 4a). By contrast, VPS28‐2‐HA levels remained unaffected (Fig. 4b). Consistent with prior findings that XBAT35 promotes degradation of the related ESCRT‐I subunit VPS23A (Yu *et al*., 2020), a parallel degradation assay confirmed gradual reduction of VPS23A with increasing XBAT35.2 (Fig. S6), validating our degradation assays performed on tobacco leaves.

**Fig. 4:**
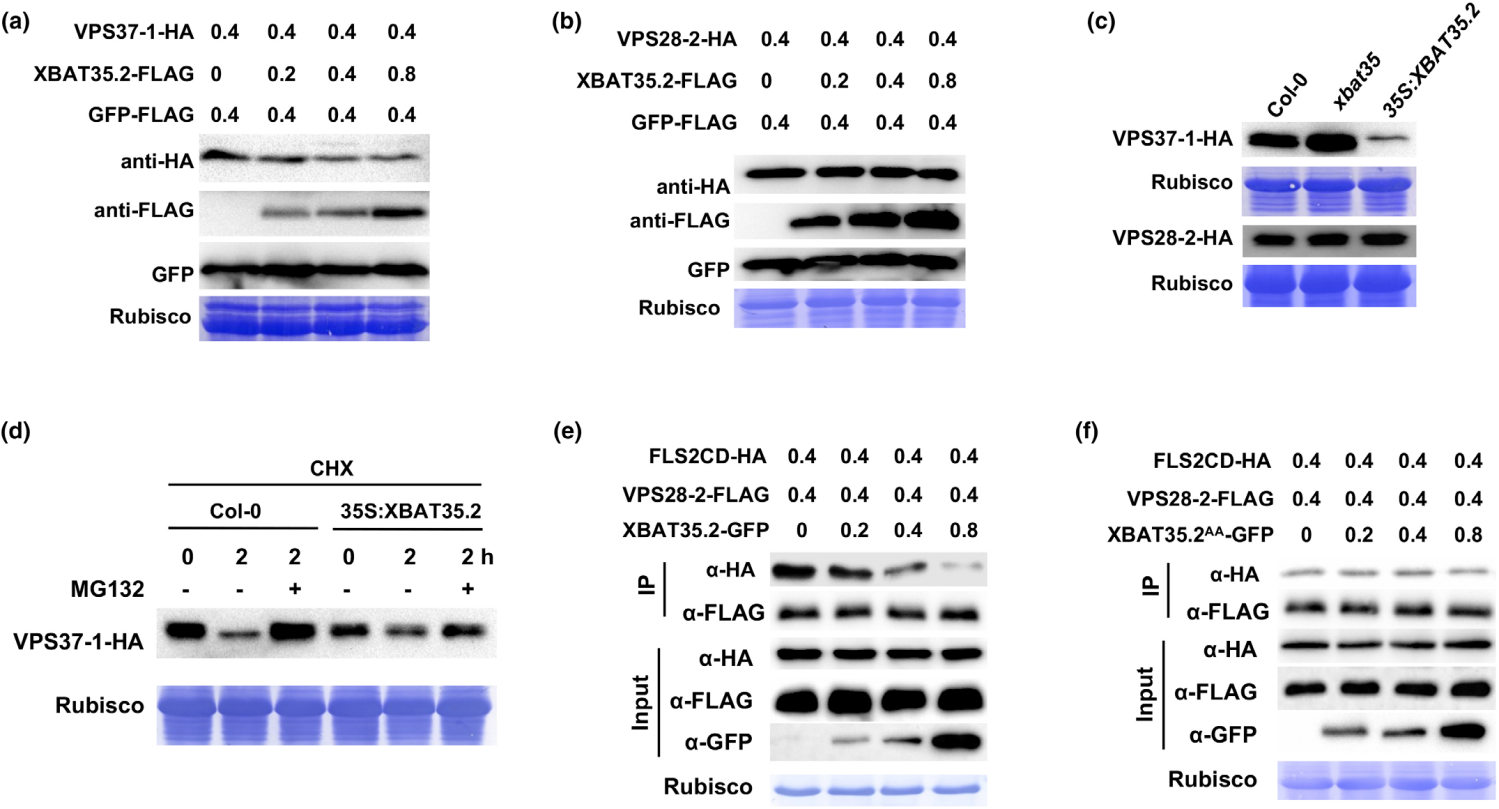
Different outcomes of VPS37‐1 and VPS28‐2 ubiquitinated by XBAT35.2 using distinct ubiquitin chains. (a, c, d) XBAT35.2 promotes the degradation of the VPS37‐1 protein in a 26S proteasome‐dependent manner. (a) *Agrobacterium tumefaciens* carrying *35S:VPS37‐1‐HA* constructs were co‐infiltrated into tobacco leaves with increasing amount of *A. tumefaciens* cells that harbor the *35S:XBAT35.2*‐*FLAG* constructs, and green fluorescent protein (GFP) was used as internal expression control. (c) The null mutation of XBAT35 blocks VPS37‐1 protein degradation whereas overexpression of XBAT35.2 significantly enhances the degradation of VPS37‐1. The transgenic seedlings where VPS37‐1‐HA proteins are expressed in the genetic background of Arabidopsis Columbia‐0 (Col‐0), *xbat35*, and *35S:XBAT35.2‐FLAG*, respectively, were grown in soil for 4 wk, followed by total proteins extraction and immunoblotting analysis. (d) MG132 treatment significantly inhibits degradation of VPS37‐1‐HA protein. Ten‐day‐old seedlings of *Arabidopsis* transgenic lines where VPS37‐1‐HA is expressed in the genetic background of Col‐0 and *35S:XBAT35.2‐FLAG* were treated with 25 μM CHX or 25 μM CHX and 75 μM MG132 as indicated for 2 h before sampling. (b, c) XBAT35.2 does not cause degradation of VPS28‐2. (b) *A. tumefaciens* carrying the *35S:VPS28‐2‐HA* construct were co‐infiltrated into tobacco leaves with increasing amount of *A. tumefaciens* cells that harbor the *35S:XBAT35.2*‐*FLAG* construct. GFP‐FLAG was used as internal expression control. (c) Neither disruption nor overexpression of XBAT35.2 affects the protein level of VPS28‐2. The transgenic seedlings where VPS28‐2‐HA proteins are expressed in the genetic background of Col‐0, *xbat35*, and 35S:XBAT35.2‐FLAG, respectively, were grown in soil for 4 wk, followed by total proteins extraction and immunoblotting analysis. (e, f) XBAT35.2 promotes the disassociation of VPS28‐2 with Flagellin Sensing 2 (FLS2), which is dependent on its E3 ligase activity. The same amount of *A. tumefaciens* cells transformed with *35S:VPS28‐2‐FLAG* and *35S:FLS2CD‐HA* were co‐infiltrated into tobacco leaves with increasing amount of *A. tumefaciens* cells that express XBAT35.2‐GFP (e) or the E3 ligase activity null mutant XBAT35.2^AA^‐GFP (f). Samples were collected 48 h after Agro‐infiltration and co‐immunoprecipitation was carried out with anti‐FLAG antibody. The immunoprecipitated samples were analyzed by immunoblotting using anti‐hemagglutinin (HA) antibody.

In stable transgenic Arabidopsis lines expressing 35S:VPS37‐1‐HA or 35S:VPS28‐2‐HA in Col‐0 (WT), *xbat35* mutant, and 35S:XBAT35.2‐FLAG backgrounds, immunoblotting revealed higher accumulation of VPS37‐1‐HA in the *xbat35* mutant compared with Col‐0, with sharply reduced levels in the 35S:XBAT35.2‐FLAG lines (Fig. 4c). This indicates that XBAT35.2 targets VPS37‐1 for degradation *in vivo*. By contrast, VPS28‐2‐HA levels were comparable across Col‐0, *xbat35*, and *35S:XBAT35.2‐FLAG* background lines (Fig. 4c), confirming K63‐linked ubiquitination of VPS28‐2 by XBAT35.2 does not lead to its degradation.

To test whether the XBAT35.2‐mediated VPS37‐1 degradation was 26S proteasome‐dependent, we performed cycloheximide (CHX) chase degradation assays using 2‐wk‐old transgenic *Arabidopsis* seedlings expressing VPS37‐1‐HA in Col‐0 and *35S:XBAT35.2‐FLAG* backgrounds, respectively. Given the low baseline VPS37‐1 levels in the 35S:XBAT35.2 lines, we used lower CHX concentrations for the seedlings of these transgenic lines. As shown in Fig. 4(d), 2‐h CHX treatment significantly reduced the amount of VPS37‐1 in both Col‐0 and *35S:XBAT35.2* plants, consistent with ongoing XBAT35.2‐mediated degradation. However, adding the proteasome inhibitor MG132 efficiently blocked this degradation. Worthy of notice, the overall VPS37‐1 levels were lower in the 35S:XBAT35.2 background than in Col‐0, likely due to constitutive XBAT35.2‐mediated ubiquitination and turnover. These results demonstrate that XBAT35.2 promotes VPS37‐1 degradation in a 26S proteasome‐dependent manner.

K63‐linked ubiquitination typically serves nonproteolytic roles, such as modulating protein activity, subcellular localization, or interaction with partners. Since VPS28‐2 is modified by XBAT35.2 via K63‐linked chains (Fig. 3c) and associates with FLS2 for endosomal sorting to MVB (Spallek *et al*., 2013), we tested whether XBAT35.2‐mediated K63‐linked ubiquitination of VPS28‐2 affects the VPS28‐2–FLS2 interaction. We co‐infiltrated equal amount of *Agrobacterium tumefaciens* harboring constructs that express VPS28‐2‐FLAG and FLS2CD‐HA protein, respectively with increasing amounts of *A. tumefaciens* carrying the XBAT35.2‐GFP constructs. After 48 h, total proteins were extracted and subjected to co‐IP assays. As shown in Fig. 4(e), FLS2CD‐HA was co‐immunoprecipitated by VPS28‐2‐FLAG, but the amount decreased progressively with increasing XBAT35.2. By contrast, the amount of FLS2CD‐HA co‐immunoprecipitated with VPS28‐2‐FLAG was unaltered when the E3 ligase‐inactive variant XBAT35.2^AA^ was used (Fig. 4f). These results suggest that XBAT35.2‐mediated K63 ubiquitination of VPS28‐2 indeed reduces the interaction of VPS28‐2 with FLS2.

### VPS37‐1 and VPS28‐2 play redundant, negative roles in FLS2‐mediated immunity by promoting vacuolar degradation of FLS2

The results obtained thus far led us to hypothesize that VPS37‐1 and/or VPS28‐2 play a negative role in FLS2‐mediated immunity, likely by promoting the vacuolar degradation of FLS2. To test this hypothesis, we identified homozygous T‐DNA insertion mutant lines, *vps37‐1.1* (CS804682) and *vps28‐2* (SALK_040274C; Fig. S7a,b), and generated the double‐mutant line *vps28‐2/vps37‐1.1* by crossing. No obvious growth or developmental defects were observed in the single and double‐mutant plants compared with the WT Col‐0 plants (Fig. S7c).

We next examined the FLS2 protein levels in these lines. In the absence of flg22 treatment, FLS2 abundance in the single mutants *vps28‐2* and *vps37‐1.1* was comparable to that in Col‐0 but was slightly elevated in the double‐mutant *vps28‐2*/*vps37‐1.1* (Fig. 5a). Upon flg22 treatment, FLS2 levels decreased markedly in Col‐0, whereas the decline was attenuated in *vps37‐1.1*, *vps28‐2* and especially in the *vps28‐2/vps37‐1.1* double mutant, which retained higher levels of FLS2 than Col‐0 or either single mutant (Fig. 5a). Conversely, in transgenic lines expressing *35S:VPS28‐2‐HA* or *35S:VPS37‐1‐HA*, FLS2 protein levels were slightly lower than in Col‐0 in the absence of flg22 (Fig. 5b). Flg22 treatment induced a reduction of FLS2 abundance in both Col‐0 and the overexpressing lines; however, the reduction was much more pronounced in plants overexpressing *VPS28‐2* or *VPS37‐1* (Fig. 5b). Because VPS28‐2 and VPS37‐1 are involved in endocytic trafficking of membrane proteins, we also examined the levels of membrane‐associated FLS2 (mFLS2) and total FLS2 (tFLS2) in plants of different genotypes (Col‐0, *xbat35*, *35S:XBAT35.2‐FLAG*, *vps28‐2/vps37‐1.1*, *35S:VPS28‐2‐HA*, and *35S:VPS37‐1‐HA*), using the *fls2* mutant as a control. As shown in Fig. 5(d), flg22 treatment for 90 min significantly reduced the proportion of membrane‐associated FLS2 (mFLS2/tFLS2) in the *xbat35* mutant, the *35S:VPS28‐2‐HA* and *35S:VPS37‐1‐HA* lines while increasing the proportion of mFLS2 in the *35S:XBAT35.2‐FLAG* and *vps28‐2/vps37‐1.1* lines, supporting the opposite roles of VPS28‐2/VPS37‐1 vs XBAT35.2 in modulating the abundance of membrane‐associated FLS2 likely through endocytic trafficking. These findings suggest VPS28‐2 and VPS37‐1 redundantly promote degradation of both nonactivated and flg22‐activated FLS2, with a more pronounced effect on activated FLS2.

**Fig. 5:**
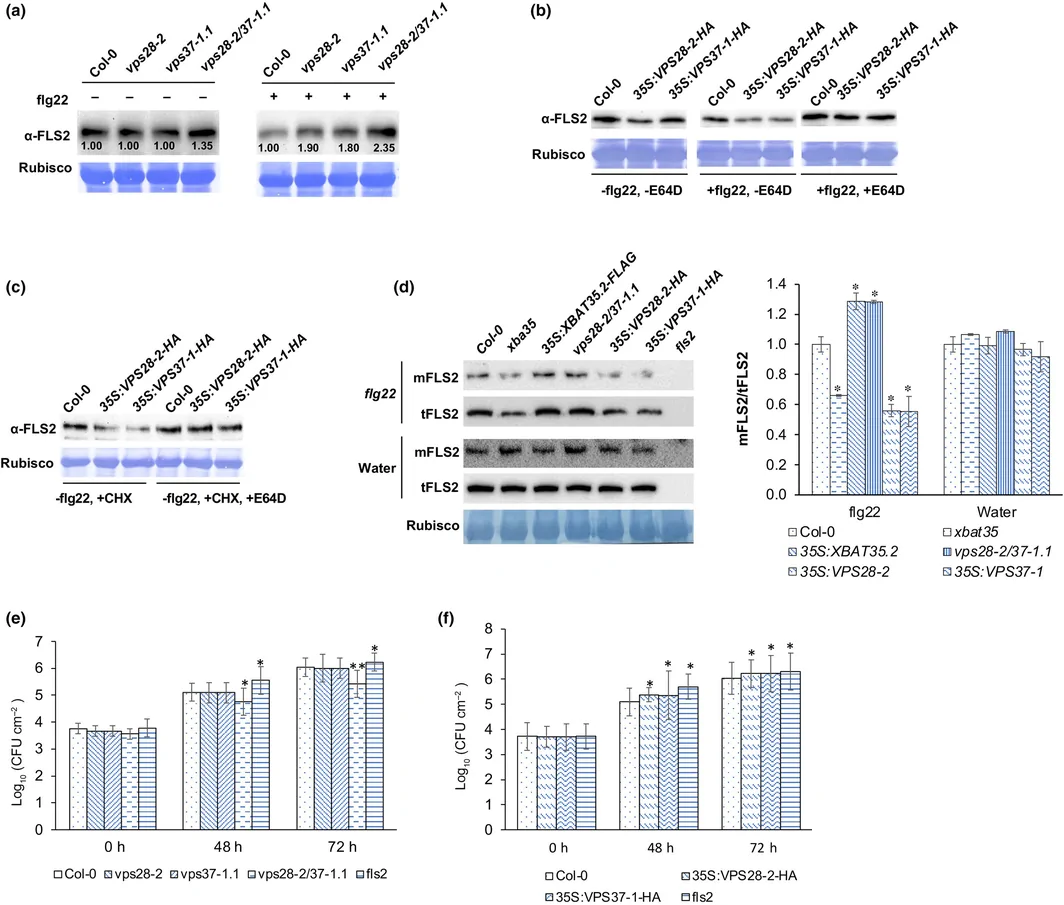
VPS28‐2 and VPS37‐1 play negative roles in immunity by promoting vacuolar degradation of Flagellin Sensing 2 (FLS2). (a) Loss of function in VPS28‐2 and VPS37‐1 redundantly inhibit flg22‐induced FLS2 degradation. Ten‐day‐old seedlings of Columbia‐0 (Col‐0) and stable transgenic lines grown on ½‐strength Murashige & Skoog (½MS) media were transferred into liquid ½MS with or without flg22 for 1 h. The numbers below the bands denote the relative abundance of the FLS2 protein, with the normalized value in untreated Col‐0 plants being set as 1. (b, c) VPS28‐2 and VPS37‐1 promote vacuolar degradation of FLS2. Ten‐day‐old seedlings of Col‐0, 35S:*VPS28‐2‐HA*, and 35S:*VPS37‐1‐HA* transgenic *Arabidopsis* plants were treated with or without flg22 and E64D (10 μg ml^−1^) for 1 h before sampling for immunoblotting analysis (b). Staining of the Rubisco indicated equal loading of the samples. VPS28‐2 and VPS37‐2 promote vacuolar degradation of non‐*de novo* synthesized, nonactivated FLS2 protein (c). Ten‐day‐old seedlings of Col‐0, 35S:*VPS28‐2‐HA*, and 35S:*VPS37‐1‐HA* transgenic *Arabidopsis* line were treated with 50 μM CHX or both 50 μM CHX and E64D (10 μg ml^−1^) before sampling for immunoblotting analysis. The CHX was used to minimize the effects of *de novo* synthesized FLS2 protein on the assay. CHX: cycloheximide, CFU: colony forming unit. (d) XBAT35.2 and VPS28‐2/VPS37‐1 differentially regulate FLS2 stability. Left panel: Four‐week‐old Arabidopsis plants of the indicated genotypes (*fls2* served as a negative control) were treated with 1 μM flg22 for 90 min, after which the levels of membrane‐associated FLS2 (mFLS2) and total FLS2 (tFLS2) were analyzed by immunoblotting using an anti‐FLS2 antibody. Sterile water treatment served as the mock control. Right panel: Band intensities of mFLS2 and tFLS2 were quantified using imagej. The normalized FLS2 signal in Col‐0 was set to 1. The ratios of mFLS2 to tFLS2 band intensities for the indicated genotypes are shown. Error bars represent the SD of three independent experiments. *, *P* < 0.05 (one‐way ANOVA followed by Tukey's honestly significant difference (HSD) test). (e, f) VPS28‐2 and VPS37‐1 redundantly modulate plant immunity as shown by pathogen growth assay on *Arabidopsis* plants in which *VPS28‐2* and *VPS37‐1* are loss‐of‐function (e) or overexpressed (f). The *fls2* mutant was included as control. For both (e, f), 4‐wk‐old plants were inoculated with *Pst* DC3000 (OD_600_ = 0.2), and the bacterial growth was determined at 48 and 72 hpi. Error bars represent the SD of nine independent samples for each genotype. *, *P* < 0.05; **, *P* < 0.01.

The ESCRT machinery plays important roles in trafficking and sorting of internalized PM proteins into MVBs for vacuolar (in yeast and plants) or lysosomal (in animals) degradation (Paez Valencia *et al*., 2016). VPS37‐1 and VPS28‐2 were previously shown to mediate flg22‐induced late endosomal trafficking of FLS2 into MVB lumens (Spallek *et al*., 2013). In the plant endocytosis and subsequent endocytic trafficking pathway, PM cargo proteins sorted into MVB are typically targeted for vacuolar degradation. Indeed, when E64D, an inhibitor of vacuolar hydrolases, was added, the degradation of FLS2 was significantly inhibited upon flg22 treatment (Fig. 5b). Importantly, CHX chase assays showed that overexpression of VPS28‐2 and VPS37‐1 also reduced FLS2 levels in the absence of flg22 elicitation, and this was similarly blocked by E64D treatment (Fig. 5c), further supporting the notion that nonactivated FLS2 proteins also undergo ESCRT‐mediated sorting for vacuolar breakdown.

Quantitative RT‐PCR (qRT‐PCR) analysis showed that expression of *XBAT35.2*, *VPS28‐2*, and *VPS37‐1* in Arabidopsis leaves was significantly induced at various timepoints following *Pst* DC3000 treatment (Fig. S8), supporting their roles in host immunity against bacterial infection. This finding is consistent with our previous report that *XBAT35.2* expression is significantly induced by flg22 treatment (Zhang *et al*., 2026). Pathogen growth assays showed comparable *Pst* DC3000 growth in single mutants *vps28‐2* and *vps37‐1.1* vs Col‐0, but significantly reduced growth in the double‐mutant *vps28‐2/vps37‐1.1* (Fig. 5e). We also performed pathogen growth assays on these knockout mutant lines and *35S:VPS28‐2‐HA* and *35S:VPS37‐1‐HA* overexpression lines that were pretreated with 1 μM flg22. Flg22 pretreatment enhanced plant immunity, with *Pst* DC3000 growth markedly lower in the *vps28‐2/vps37‐1.1* double mutant (Fig. S9a). By contrast, the growth of the *Pst* DC3000 Δ*fliC* mutant strain that lacks flagellin (Kvitko *et al*., 2009) was similar across the WT Col‐0, *vps37‐1.1*, *vps28‐2*, *vps28‐2/vps37‐1.1*, and fls2 mutant lines (Fig. S9b). These results suggested negative roles of VPS28‐2 and VPS37‐1 in FLS2‐mediated immunity against *Pst* DC3000. Consistently, overexpression of *VPS28‐2* or *VPS37‐1* increased host susceptibility to *Pst* DC3000 (Figs 5f, S9c,d).

Collectively, these findings demonstrate that VPS37‐1 and VPS28‐2 play redundant, negative roles in FLS2‐mediated immunity by promoting vacuolar degradation of FLS2.

### VPS28‐2, VPS37‐1, and XBAT35.2 modulate downstream FLS2‐mediated immune responses without affecting the early ROS burst

To gain a deeper understanding of the roles of VPS28‐2, VPS37‐1, and XBAT35.2 in FLS2‐mediated immunity, we further examined their effects on specific immune responses using loss‐of‐function mutants and overexpression lines. Interestingly, flg22‐induced ROS production was comparable among Col‐0, *xbat35*, 35S:*XBAT35.2*, *vps28‐2*, *vps37‐1.1*, the *vps28‐2/vps37‐1.1* double mutant, 35S:*VPS28‐2*, and 35S:*VPS37‐1* lines at all time points examined (Fig. 6a). This result aligns with the rapid kinetics of the ROS burst, which occurs before ligand‐induced endocytic trafficking of FLS2 is initiated (Robatzek *et al*., 2006) and is consistent with a previous report (Mbengue *et al*., 2016). By contrast, flg22‐induced activation of mitogen‐activated protein kinases 3 and 6 (MAPK3 and MAPK6) was enhanced in the 35S:*XBAT35.2* line and the *vps28‐2/vps37‐1.1* double mutant but significantly reduced in the *xbat35* and 35S:*VPS28‐2*, and 35S:*VPS37‐1* lines compared with Col‐0 (Fig. 6b). These differences became more obvious at 60 min and were particularly pronounced 90 min after flg22 treatment.

**Fig. 6:**
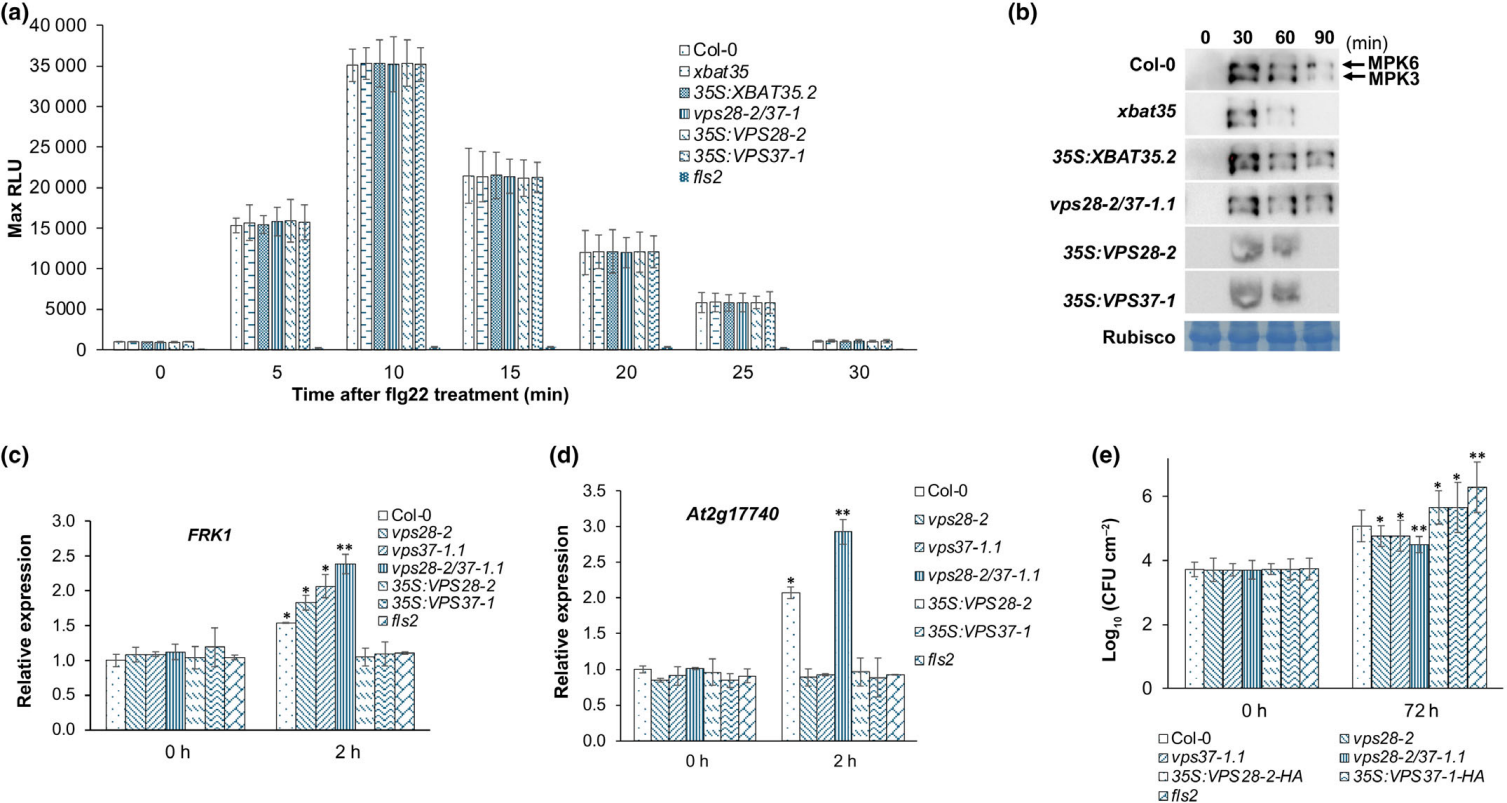
Immune responses of Arabidopsis Columbia‐0 (Col‐0), *xbat35*, 35S:*XBAT35.2*, *vps28‐2*, *vps37‐1.1*, *vps28‐2/vps37‐1.1* double mutant, 35S:*VPS28‐2*, and 35S:*VPS37‐1* lines after flg22 treatment. (a) Reactive oxygen species (ROS) production in Arabidopsis genotypes with altered *XBAT35.2*, *VPS28‐2*, or *VPS37‐1* expression. ROS production was measured using leaf disks from plants of the indicated genotypes. The *fls2* mutant served as a negative control. The residual ROS signal detected in the *fls2* mutant likely represents Flagellin Sensing 2 (FLS2)‐independent and/or background ROS production. Error bars represent the SD of eight leaf disks (*n* = 8) from three replicates. No significant differences were detected among the genotypes by one‐way ANOVA followed by Tukey's honestly significant difference (HSD) test. RLU, Relative Luminescence Units. (b) Immunoblot analysis of flg22‐induced activation of MAPK3 and MAPK6. Four‐week‐old Arabidopsis plants were treated with 1 μM flg22, and leaf samples were collected at 0, 30, 60, and 90 min after treatment. Activated MPK3 and MPK6 were detected using an anti‐phospho‐p44/42 MAPK (Erk1/2) antibody. (c, d) Quantitative reverse transcription polymerase chain reaction (qRT‐PCR) analysis of *FRK1* (c) and *At2g17740* (d) expression in Arabidopsis leaves pretreated with 1 μM flg22. Error bars represent the SD of nine individual plants for each genotype. *, *P* < 0.05; **, *P* < 0.01 (one‐way ANOVA followed by Tukey's HSD test). (e) Pretreatment with flg22 enhanced the suppression of *Pst* DC3000 growth in *VPS28‐2* and/or *VPS37‐1* loss‐of‐function lines and enhanced bacterial growth in the corresponding overexpression lines. Four‐week‐old Arabidopsis plants were pretreated with 1 μM flg22 for 2 h, followed by spray inoculation with *Pst* DC3000 (OD₆₀₀ = 0.2). Error bars represent the SD of nine independent biological samples for each genotype. *, *P* < 0.05; **, *P* < 0.01 (one‐way ANOVA followed by Tukey's HSD test). CFU, colony forming unit.

We next measured the transcript levels of two PAMP‐responsive early defense genes, *FRK1* and *At2g17740*, in Col‐0 and in *VPS28‐2*‐ and *VPS37‐1*‐overexpressing and loss‐of‐function lines. Both genes are known to be strongly induced by *Pst* DC3000 infection and flg22 treatment (Asai *et al*., 2002; He *et al*., 2006). Consistent with these previous reports, the expression of both genes was induced by flg22 treatment in Col‐0 (Fig. 6c,d). However, flg22‐induced *FRK1* expression was further enhanced in the *vps28‐2* and *vps37‐1.1* single mutants as well as the *vps28‐2/vps37‐1.1* double mutant, whereas *At2g17740* expression was significantly higher than in Col‐0 only in the *vps28‐2/vps37‐1.1* double mutant. By contrast, flg22 failed to induce either gene in the *VPS28‐2*‐ or *VPS37‐1*‐overexpressing lines. Finally, we evaluated the effect of flg22 pretreatment on bacterial growth in VPS28‐2 and VPS37‐1 loss‐of‐function and overexpression lines. As shown in Fig. 6(e), flg22 pretreatment enhanced *Pst* DC3000 growth in 35S:*VPS28‐2*, and 35S:*VPS37‐1* lines but further suppressed bacterial growth in the *VPS28‐2* and/or *VPS37‐1* loss‐of‐function lines compared with Col‐0. Notably, suppression of *Pst* DC3000 growth was not observed in the *vps28‐2* or *vps37‐1.1* single mutants in the absence of flg22 pretreatment (Fig. 5e).

### XBAT35.2 modulates FLS2 protein level via VPS28‐2 and VPS37‐1 in flg22‐ and *Pst* DC3000‐triggered plant immunity

We have so far shown that VPS37‐1 and VPS28‐2 are involved in flg22 and *Pst* DC3000‐triggered plant immunity by promoting FLS2 turnover (Figs 5, 6), whereas XBAT35.2 positively regulates immunity by stabilizing FLS2 (Zhang *et al*., 2026). We also demonstrated that XBAT35.2 interacts with and modifies VPS37‐1 and VPS28‐2 using distinct ubiquitin linkage types (Fig. 3), resulting in different outcomes for VPS37‐1 and VPS28‐2 (Fig. 4). Collectively, these findings suggest that XBAT35.2 and VPS37‐1/VPS28‐2 likely function in the same pathway but with opposing effects on FLS2‐mediated immunity. To test this hypothesis, we generated homozygous transgenic lines overexpressing *35S:VPS28‐2‐HA* and *35S:VPS37‐1‐HA* in the *xbat35* and Col‐0 backgrounds for exploring the genetic relationship of XBAT35.2 and VPS37‐1/VPS28‐2. We examined FLS2 protein levels in these lines using Col‐0 and *xbat35* as control (Fig. 7a). Without flg22 treatment, overexpression of VPS28‐2 or VPS37‐1 slightly reduced FLS2 abundance, with a marginally greater reduction in the *xbat35* background than in Col‐0 (Fig. 7a, upper panel). Upon flg22 activation, FLS2 levels decreased in all lines, but the reduction was more pronounced in VPS28‐2‐ or VPS37‐1‐overexpressing lines. Notably, FLS2 reduction was most significant in the *xbat35* background overexpressing VPS28‐2 or VPS37‐1 (Fig. 7a, lower panel). These results, together with prior findings mentioned above, indicate that VPS28‐2 and VPS37‐1 are epistatic to XBAT35.2 in modulating FLS2 protein level during flg22 and *Pst* DC3000‐triggered plant immunity, with XBAT35.2 regulating FLS2 level via VPS28‐2 and VPS37‐1.

**Fig. 7:**
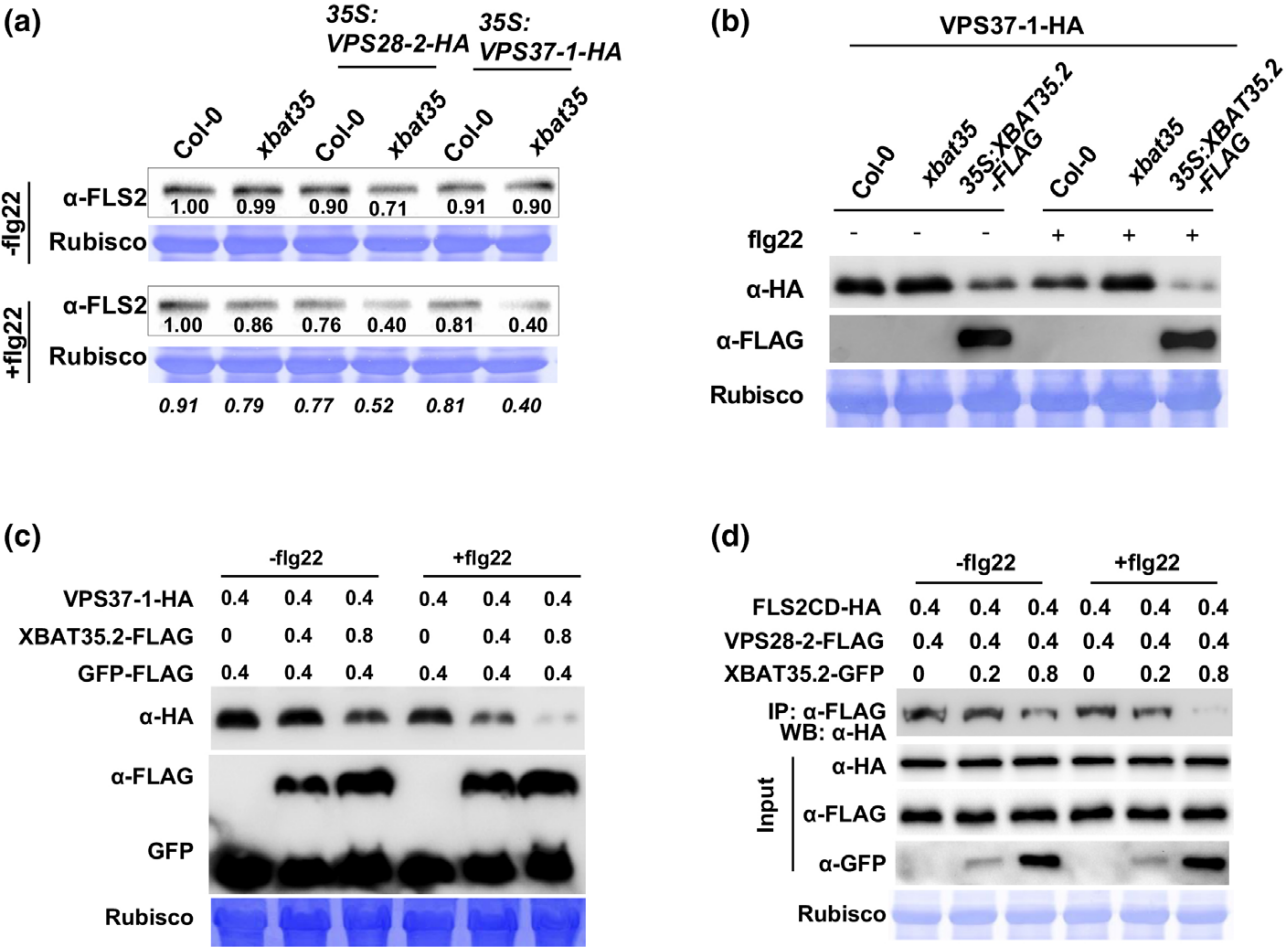
Arabidopsis XBAT35.2 regulates Flagellin Sensing 2 (FLS2)‐mediated plant immunity via VPS28‐2 and VPS37‐1. (a) The FLS2 protein level in Columbia‐0 (Col‐0), *xbat35*, *35S:VPS28‐2‐HA*/Col‐0, 35S*:VPS28‐2‐HA*/*xbat35*, 35S*:VPS37‐1‐HA*/*Col‐*0, and *35S:VPS37‐1‐HA*/*xbat35* transgenic lines in the absence or presence of flg22 treatment. Ten‐day‐old seedlings were treated with 1 μM flg22 for 1 h before sampling for protein extraction. The numbers below the bands denote the relative abundance of the FLS2 protein, with the normalized value in untreated and treated Col‐0 plants being set as 1. The italicized numbers at the bottom denote the ratio of imagej‐quantified and normalized relative abundance of the FLS2 in flg22‐treated sample compared with the corresponding non‐flg22‐treated sample for each genotype. (b) Flg22 treatment enhances XBAT35.2‐mediated degradation of VPS37‐1 in *Arabidopsis*. Ten‐day‐old seedlings of the *Arabidopsis* transgenic line where VPS37‐1‐HA is expressed in the genetic background of Col‐0, *xbat35*, and *35S:XBAT35.2‐FLAG* were treated with or without flg22 for 1 h before sampling for immunoblotting analysis. (c) Flg22 treatment enhances XBAT35.2‐mediated degradation of VPS37‐1 in tobacco leaves. The same amount of *Agrobacterium tumefaciens* cells expressing VPS37‐1‐HA or GFP‐FLAG were co‐infiltrated into tobacco leaves with increasing amount of *A. tumefaciens* cells that carry the *35S:XBAT35.2‐FLAG* constructs. Tobacco leaves were sampled 3 d after Agro‐infiltration. For flg22 treatment, 1 μM flg22 was infiltrated into Agro‐infiltrated tobacco leaves 0.5 h before sampling. (d) Flg22 treatment enhances XBAT35.2‐mediated disassociation of VPS28‐2 with FLS2. The same amount of *A. tumefaciens* cells transformed with *35S:VPS28‐2‐FLAG* and *35S:FLS2CD‐HA* were co‐infiltrated into tobacco leaves with increasing amount of *A. tumefaciens* cells that express XBAT35.2‐GFP. Samples were collected 3 d after Agro‐infiltration and co‐immunoprecipitation was carried out with anti‐FLAG antibody. The immunoprecipitated samples were analyzed by immunoblotting using anti‐HA antibody. For flg22 treatment, 1 μM flg22 was injected into Agro‐infiltrated tobacco leaves 0.5 h before sampling.

To further corroborate these findings, we examined the stability of VPS37‐1‐HA under flg22 treatment in transgenic lines expressing VPS37‐1‐HA in Col‐0, *xbat35*, and 35S:XBAT35.2‐FLAG backgrounds. One‐hour flg22 treatment reduced VPS37‐1‐HA levels in all three genotypes (Fig. 7b). However, the decline was markedly attenuated in *xbat35* compared with Col‐0, whereas VPS37‐1‐HA became nearly undetectable in XBAT35.2‐OEX lines (Fig. 7b). Degradation assays performed in tobacco leaves further showed that flg22 treatment accelerated XBAT35.2‐mediated VPS37‐1 degradation (Fig. 7c). Taking with the data shown in Fig. 4, these results demonstrate that flg22 treatment enhances VPS37‐1 degradation and that elevated XBAT35.2 significantly enhances this process.

Since XBAT35.2‐mediated K63‐ubiquitination of VPS28‐2 does not affect its stability but instead significantly reduces its association with FLS2 (Fig. 4), we analyzed the effect of XBAT35 on the interaction of VPS28‐2 with FLS2 in flg22‐triggered plant immunity by Co‐IP assay. Equal amounts of *A. tumefaciens* harboring constructs that express VPS28‐2‐FLAG and FLS2CD‐HA were co‐infiltrated with increasing amounts of *A. tumefaciens* carrying the XBAT35.2‐GFP constructs. As shown in Fig. 7(d), the amount of FLS2CD‐HA co‐immunoprecipitated with VPS28‐2‐FLAG decreased progressively with increasing XBAT35.2, and flg22 treatment further amplified this reduction. These results collectively confirm that XBAT35.2 modulates FLS2 protein levels via VPS28‐2 and VPS37‐1 in FLS2‐mediated plant immunity.

## Discussion

Through K63 and K48‐linked ubiquitination, respectively, XBAT35.2 modifies two different subunits of the ESCRT‐I complex, VPS28‐2 and VPS37‐1, which we show in this study mediate the vacuolar degradation of FLS2. Via this mechanism, XBAT35.2 positively regulates the FLS2 protein levels and, consequently, flg22‐ and bacterial pathogens‐triggered plant immunity. Activated FLS2 protein was previously reported to be targeted by PUB12/PUB13 for ubiquitination and subsequent 26S proteasome‐dependent degradation, thereby attenuating host immune signaling (Lu *et al*., 2011). In the presence of flg22, BAK1 phosphorylates the E3 ubiquitin ligases PUB12 and PUB13, which then interact with FLS2 and tag it with ubiquitin chains. This modification marks FLS2 for proteasomal degradation, effectively dampening FLS2‐mediated immune signaling. Our findings thus reveal an additional layer of regulation for fine‐tuning FLS2 protein levels by modulating subunits of the ESCRT‐I complex (Fig. 8). Whether the two regulatory circuits for control of FLS2 abundance are connected – and if so, how they are coordinated – represent interesting questions to be addressed in future experiments.

**Fig. 8:**
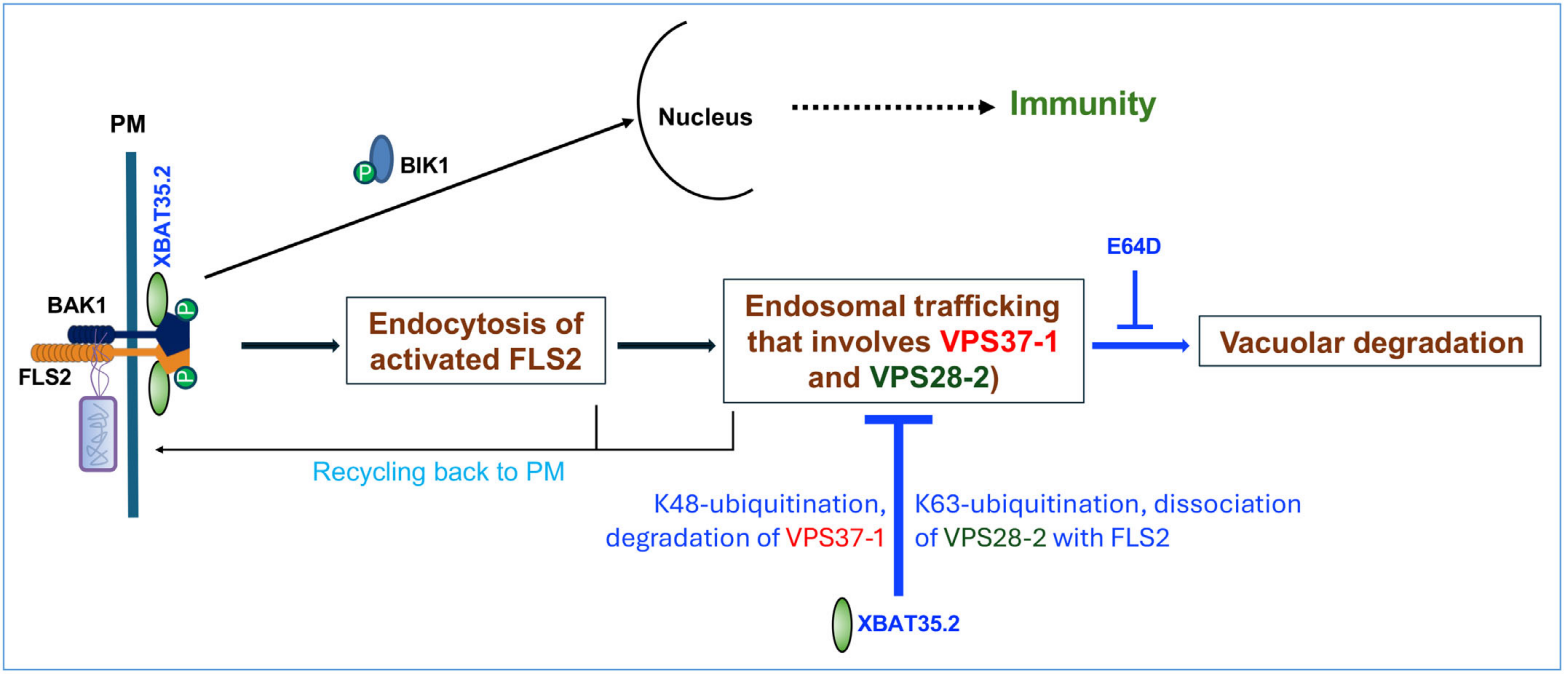
Working model depicting the positive regulation of Flagellin Sensing 2 (FLS2)‐mediated plant immunity by XBAT35.2 through modifying the ESCRT‐I subunits VPS28‐2 and VPS37‐1 with distinct ubiquitin chain types. Upon *Pseudomonas syringae* pv *tomato* (*Pst*) infection, Arabidopsis FLS2 recognizes and binds to bacterial flagellin, leading to the activation of FLS2 and immune signaling that culminates in plant immunity. Bacterial pathogen infection enhances the association of XBAT35.2 with FLS2 and BAK1 but promotes the dissociation between XBAT35.2 and BIK1 (Zhang *et al*., 2026). The activated FLS2 undergoes endocytosis and is targeted for vacuolar breakdown (Choi *et al*., 2013). The degradation of FLS2 at the vacuole is inhibited by E64D, an exogenous inhibitor of lysosomal/vacuolar hydrolases. XBAT35.2 sabotages VPS28‐2/VPS37‐1‐mediated vacuolar degradation of FLS2 by catalyzing attachment of K48 and K63‐linked polyubiquitin chains to VPS37‐1 and VPS28‐2, respectively, which leads to proteasomal degradation of VPS37‐1 and disassociation of VPS28‐2 from FLS2. By doing so, XBAT35.2 effectively stabilizes FLS2 and promotes host plant immunity against bacterial pathogens. The thick arrows indicate endocytosis and endosomal trafficking steps reported previously (in black) and in the present study (in blue). The blue color text, arrow, and ‘T’ shape symbols mark findings obtained in this study. The thin solid and dashed black arrows indicate previously reported signaling steps of FLS2‐mediated immunity. ‘T’ shape and upside‐down ‘T’ shape symbols denote inhibition.

Given the involvement of the ESCRT machinery in a wide array of cellular and physiological pathways, cells must maintain tight homeostasis of its components to support diverse functions. K63‐linked ubiquitination of VPS28‐2 reduces its association with FLS2 without inducing VPS28‐2 protein turnover. This selective modification likely preserves VPS28‐2 availability for assembly into the ESCRT‐I complex, which is crucial for many other cellular functions. In this context, the distinct ubiquitination of VPS28‐2 and VPS37‐1 directed by XBAT35.2 may represent a self‐protective mechanism that safeguards overall ESCRT‐I functionality while modulating specific pathways. Concurrently, modification of these two ESCRT‐I subunits enables fine‐tuning the ESCRT‐I‐mediated sorting of internalized FLS2 for vacuolar degradation, which is critical for precise control of the abundance of FLS2 at the PM.

Nonactivated FLS2 proteins that undergo endocytosis can be recycled to the PM through EEs or enter Brefeldin A (BFA)‐sensitive endosomal pathway (Beck *et al*., 2012). Our results show that elevated XBAT35.2 levels increase FLS2 protein in the absence of flg22 treatment (Fig. 7b; Zhang *et al*., 2026), suggesting that a portion of the internalized nonactivated FLS2 proteins may be sorted by the ESCRT machinery for vacuolar degradation. Consistent with this, overexpression of VPS28‐2 and VPS37‐1 reduces FLS2 protein levels in the absence of flg22 treatment (Fig. 5a,c), whereas the FLS2 protein level is slightly higher in the *vps28‐2/vps37‐1.1* double mutant than in Col‐0 WT plants (Fig. 5a). Furthermore, the flg22‐induced decrease in FLS2 is significantly attenuated in the *vps28‐2/vps37‐1.1* double mutant compared with Col‐0 and *vps28‐*2 and vps37*‐1.1* single mutants (Fig. 5a). Treatment with E64D inhibits the vacuolar degradation of both no‐activated and activated FLS2 (Fig. 5b,c). These observations support the notion that both nonactivated and activated FLS2 undergo ESCRT‐assisted targeting for vacuole‐mediated degradation, and that flg22‐triggered activation of immune signaling enhances ESCRT‐I‐mediated vacuolar degradation of FLS2. Nonactivated and activated FLS2 have previously been shown to enter different endosomal pathways (Beck *et al*., 2012). It will be intriguing to determine how these two forms of FLS2 are targeted by the ESCRT machinery during endosomal trafficking.

The ESCRT machinery include three subcomplexes in plants – ESCRT‐I, ESCRT‐II, and ESCRT‐III – and plays a central role in sorting internalized PM proteins for vacuolar destruction (Paez Valencia *et al*., 2016; Vietri *et al*., 2020). Over the past four decades, extensive studies have elucidated the composition, structure, assembly mechanisms, and functions of the ESCRT machinery. By contrast, relatively little is known about how components of the ESCRT complex are modulated to function in diverse pathways. In mammals, tumor suppressor gene101 (Tsg101), a key ESCRT‐I component, has been shown to be multi‐monoubiquitinated or polyubiquitinated (Ferraiuolo *et al*., 2020). However, direct evidence that modifications of Tsg101 by ubiquitin affect trafficking and sorting of cell‐surface receptors by the ESCRT‐I machinery remains limited. In plants, VPS23A, the Tsg101 homolog and FREE1 are essential for MVB biogenesis and the endosome‐to‐vacuole sorting of membrane receptors (Spitzer *et al*., 2006; Gao *et al*., 2014). FREE1 has been identified as a crucial regulator in plant response to iron deficiency stress, and its homeostasis is controlled by the RING‐finger E3 ubiquitin ligases, SINA of *Arabidopsis thaliana* (SINATs; Xiao *et al*., 2020). In addition, FREE1 and VPS23A are involved in modulating ABA signaling by transporting and sorting the ABA receptors PYR1/PYL4 for vacuole‐mediated turnover (Belda‐Palazon *et al*., 2016; Yu *et al*., 2016). Both FREE1 and VPS23A are also targeted by SINAT family E3 ubiquitin ligases, which promote ABA signaling (Xia *et al*., 2020). VPS23A has further been shown to be targeted by XBAT35 for proteasome‐mediated degradation during ABA signaling regulation (Yu *et al*., 2020).

In the present study, we demonstrate that the ubiquitin E3 ligase XBAT35.2 modifies VPS37‐1 and VPS28‐2, two subunits of the ESCRT‐I complex, through K48‐ and K63‐linked ubiquitination, respectively. These modifications promote 26S proteasome‐mediated degradation of VPS37‐1 and reduce the interaction between VPS28‐2 and FLS2. Interestingly, the rice ANK‐RING type ubiquitin E3 ligase XB3 has been shown to interact with the rice cell‐surface immune receptor XA21, and reduced XB3 levels result in decreased XA21 abundance and compromised resistance to the avirulent race of *Xanthamonas oryzae* pv *oryzae* (Wang *et al*., 2006). However, the mechanism by which XB3 regulates XA21 abundance remains unclear. Based on our findings, we speculate that endocytosis and ensuing trafficking and sorting processes contribute to the regulation of XA21 protein levels, potentially through XB3‐mediated targeting of the ESCRT machinery to modulate XA21 abundance at the PM. More recently, the RING type E3 SGD1 and its homologs, which regulate grain yield in *Setaria italica* and other crops, was shown to ubiquitinate and stabilize the brassinosteroid receptor BRI1 (Tang *et al*., 2023). The authors proposed that this stabilization of BRI1 by SGD1 may occur through modulation of the endocytosis pathway.

Cell‐surface receptors play essential roles in perceiving and transmitting extracellular signals to intracellular processes. Together with previous reports on VPS23A and FREE1, our results suggest that modification of ESCRT machinery components by ubiquitin could be a widespread regulatory mechanism for cellular signaling, likely due to the central role of the ESCRT machinery in controlling the turnover of membrane proteins, including the numerous cell‐surface receptors.

## Competing interests

None declared.

## Author contributions

CW designed and performed vast majority of the experiments, analyzed data, and wrote and edited the manuscript. YZ built some constructs for the BiFC assays. BZ helped perform some of the *in vitro* ubiquitination assays. PV built constructs for purifying the AtUBC13 and AtUEV1a proteins. LZ designed experiments, analyzed data, wrote and edited the manuscript.

## Disclaimer

The New Phytologist Foundation remains neutral with regard to jurisdictional claims in maps and in any institutional affiliations.

## Supporting information


**Dataset S1** Arabidopsis and *Nicotiana benthamiana* VPS28‐2 Protein Sequence Alignment.
**Dataset S2** Arabidopsis and *Nicotiana benthamiana* VPS37‐1 Protein Sequence Alignment.
**Dataset S3** Visualization and validation of *Nicotiana benthamiana* NbVPS37‐1, NbVPS28‐2, and NbVPS23a sequences using Scaffold 5.
**Dataset S4** Visualization and validation of Arabidopsis XBAT35.2, VPS28‐2, and VPS37‐1 sequences using Scaffold 5.
**Fig. S1** *Nicotiana Benthamiana* and Arabidopsis VPS28‐2 and VPS37‐1 were co‐immunoprecipitated with XBAT35.2 in the co‐IP/LC‐MS/MS assay.
**Fig. S2** XBAT35.2 does not interact with DRP1A and DRP2B.
**Fig. S3** *Pst* DC3000 infection enhances the interaction of XBAT35.2 with VPS28‐2 and VPS37‐1.
**Fig. S4** XBAT35.2 does not ubiquitinate VPS37‐1 with K63‐linked ubiquitin chains.
**Fig. S5** XBAT35.2 directs ubiquitination of VPS28‐2 and VPS37‐1 *in vivo*.
**Fig. S6** XBAT35.2 promotes degradation of VPS23a.
**Fig. S7** Identification of the Arabidopsis *vps28‐2* and *vps37‐1.1* T‐DNA insertion mutants.
**Fig. S8** Expression of *XBAT35.2*, *VPS28‐2* and *VPS37‐1* is induced following *Pst* DC3000 treatment.
**Fig. S9** Bacterial pathogen growth in *VPS28‐2* and *VPS37‐1* loss‐of‐function and overexpression lines.
**Table S1** Primers used for genotyping, cloning, qRT‐PCR, and other experiments for this study.
**Table S2** Representative plant immunity‐ and ESCRT‐related proteins co‐immunoprecipitated with XBAT35.2‐FLAG in the Co‐IP/LC‐MS/MS assays.
Please note: Wiley is not responsible for the content or functionality of any Supporting Information supplied by the authors. Any queries (other than missing material) should be directed to the *New Phytologist* Central Office.

## Data Availability

All data generated or analyzed during this study are included in this article and its Supporting Information file (Datasets S1–S4; Figs S1–S9; and Tables S1, S2). Protein sequence data for the Arabidopsis genes used in this research can be found in the GenBank/EMBL libraries under the following accession nos.: FLS2: Q9FL28, BAK1: AEE86224, BIK1: O48814, XBAT35.2: OAP01869, DRP1A: P42697, DRP2B: Q9LQ55, VPS37‐1: Q9SCP9, and VPS28‐2: Q9S9T7. The sequence of *Nicotiana benthamiana* VPS37‐1 and VPS28‐2 proteins can be found at the *N. benthamiana* and *N*. *tabacum* Omics database, http://lifenglab.hzau.edu.cn/Nicomics/index.php (Wang *et al*., 2024): NbVPS28‐2.1: Nbe03g32210, NbVPS28‐2.2: Nbe10g10130, NbVPS28‐2.3: Nbe17g20060, NbVPS28‐2.4: Nbe18g25060, NbVPS37‐1.1: Nbe10g05990, NbVPS37‐1.2: Nbe13g03380, and NbVPS37‐1.3: Nbe15g09500.
