# Supplementary material for: Differential ubiquitin modifications of ESCRT‐I subunits VPS37‐1 and VPS28‐2 positively regulate plant immunity
Source: New Phytol. 2026 Sep 29;252(5):1988–2005. doi: 10.1111/nph.71590 (PMC13738685; doi:10.1111/nph.71590)
Supplement: Supplementary file 1 [file NPH-252-1988-s001.pdf]

New Phytologist Supporting Information

Differential Ubiquitin Modifications of ESCRT-I Subunits VPS37-1 and VPS28-2 Positively Regulate Plant Immunity

Chaofeng Wang, Yi Zhang, Bangjun Zhou, Pooja Verma, and Lirong Zeng

Accepted: September 1, 2026

Supplementary Figures

a

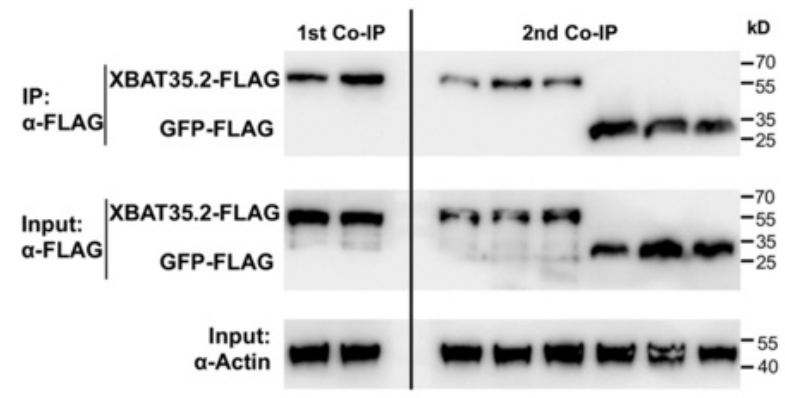

b

IP/LC-MS/MS

Protein	Unique peptide count					
	GFP-FLAG			XBAT35.2-FLAG		
GFP	29	29	27	0	0	0
XBAT35.2	0	0	0	2	5	3
VPS28-2 (Nbe10g10130.1)	0	0	0	2	0	0
VPS37-1 (Nbe10g05990.1)	0	0	0	0	2	0
VPS23A (Nbe13g03040.1)	0	0	0	10	10	11
VPS23A (Nbe08g03450.1)	0	0	0	10	10	11

c

IP/LC-MS/MS

Protein	Unique peptide count					
	Col-0			XBAT35.2-FLAG		
XBAT35.2 (AT3G23280.2)	0	0	0	28	28	29
VPS28-2 (AT4G05000)	0	0	0	14	14	14
VPS37-1 (AT3G53120)	0	0	0	17	15	17

Figure S1. The *N. Benthamiana* and Arabidopsis VPS28-2 and VPS37-1 were co-immunoprecipitated with XBAT35.2 in the co-IP/LC-MS/MS assay

(a) Detection of the expression of the bait proteins XBAT35.2-FLAG and GFP-FLAG with Co-IP (IP and Input) samples before LC-MS/MS. A. tumefaciens cells harboring the constructs that express XBAT35.2-FLAG or GFP-FLAG were separately infiltrated into tobacco leaves. Forty-eight hours post-infiltration, the leaves were treated with 1μM flg22 for one hour. Samples were then collected, and total proteins were extracted followed by Co-IP carried out with anti-FLAG antibody, and the immuno-precipitated and Input samples were analyzed by immunoblotting using anti-FLAG antibody.

(**b**) Major ECSTR-I components, NbVPS28-2, NbVPS37-1, and NbVPS23a, were identified by IP/LC–MS/MS using Arabidopsis XBAT35.2 as bait. *Agrobacterium tumefaciens* cells carrying XBAT35.2-FLAG or GFP-FLAG constructs were infiltrated into tobacco leaves. At 48 h post-infiltration, leaves were treated with 1 μM flg22 for 1 h before sample collection. Total proteins were extracted, and IP was performed using an anti-FLAG antibody, followed by LC–MS/MS analysis. For each group (XBAT35.2-FLAG or GFP-FLAG), three biological replicates were prepared. MG132 (75 μM) was included during IP sample preparation.

(**c**) Arabidopsis ECSTR-I core components, VPS28-2 and VPS37-1, were identified by IP/LC–MS/MS using Arabidopsis XBAT35.2 as bait. Total proteins were extracted from leaf tissue of XBAT35.2-FLAG overexpression transgenic line and Col-0 wild type plants treated with 1 μM flg22 for 1 h before sample collection. Total proteins were extracted and IP was performed using an anti-FLAG antibody, followed by LC–MS/MS analysis. For each group (35S:XBAT35.2-FLAG or Col-0), three biological replicates were prepared. MG132 (75 μM) was included during IP sample preparation.

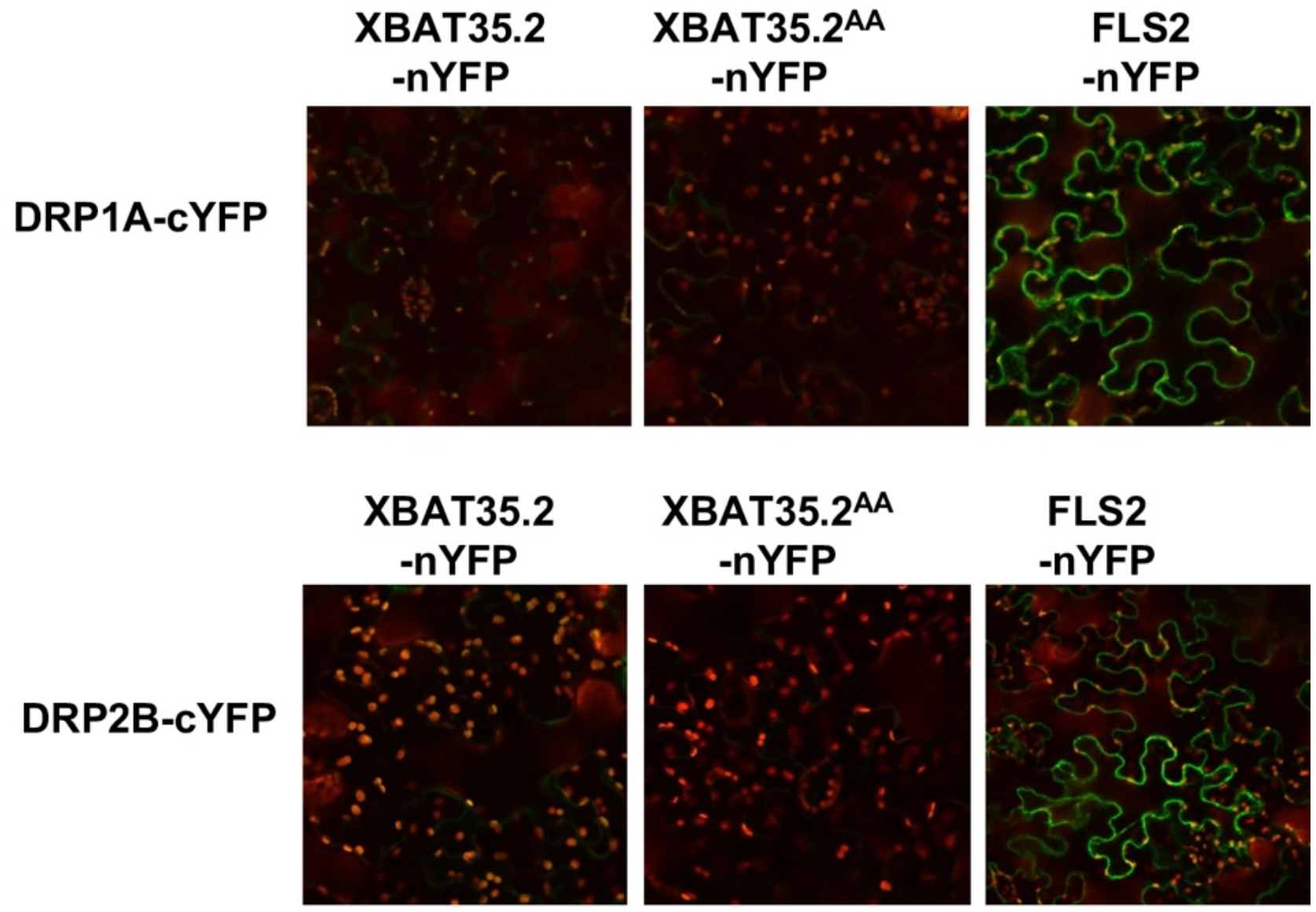

Figure S2. Arabidopsis XBAT35.2 does not interact with DRP1A and DRP2B.

The interaction of cYFP-fused DRP1A and DRP2B with nYFP-fused XBAT35.2, and its E3 ligase activity null mutant (XBAT35.2^{AA}) was examined by BiFC in tobacco leaf epidermal cells. YFP signal (pseudo-colored in green) indicates interaction between the two proteins tested. The tobacco leaves were collected and examined on the third day after Agro-infiltration. FLS2 was used as positive control.

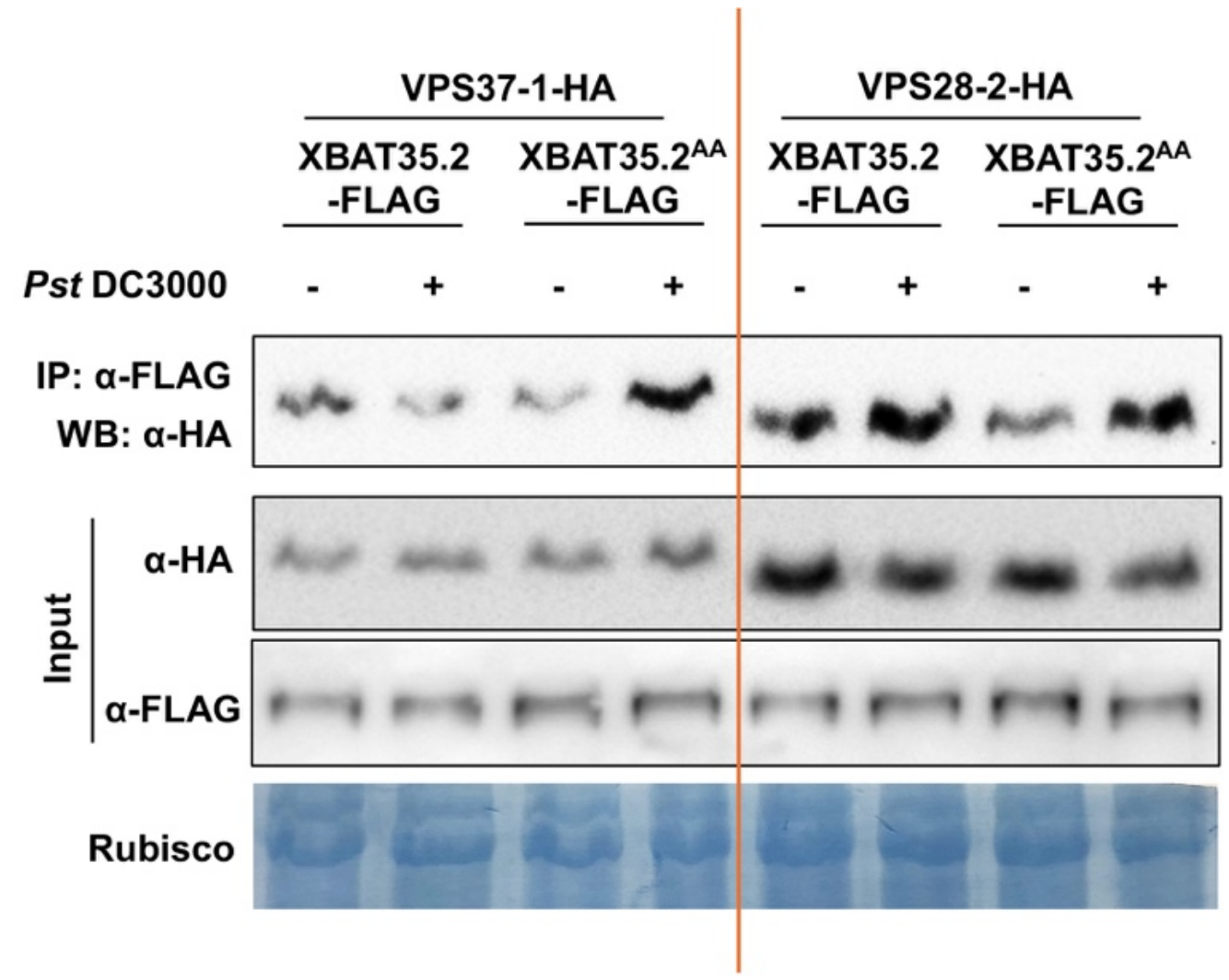

Figure S3 *Pst* DC3000 infection enhances the interaction of XBAT35.2 with VPS28-2 and VPS37-1. *Agrobacterium tumefaciens* cells carrying constructs expressing XBAT35.2-FLAG or XBAT35.2^{AA}-FLAG together with VPS28-2-HA or VPS37-1-HA were co-infiltrated into *Nicotiana benthamiana* leaves. Three days after agroinfiltration, the leaves were inoculated with *Pst* DC3000 for 1 h before sample collection. Total proteins were extracted and subjected to co-immunoprecipitation (Co-IP) using an anti-FLAG antibody. The immunoprecipitated proteins were subsequently analyzed by immunoblotting with an anti-HA antibody.

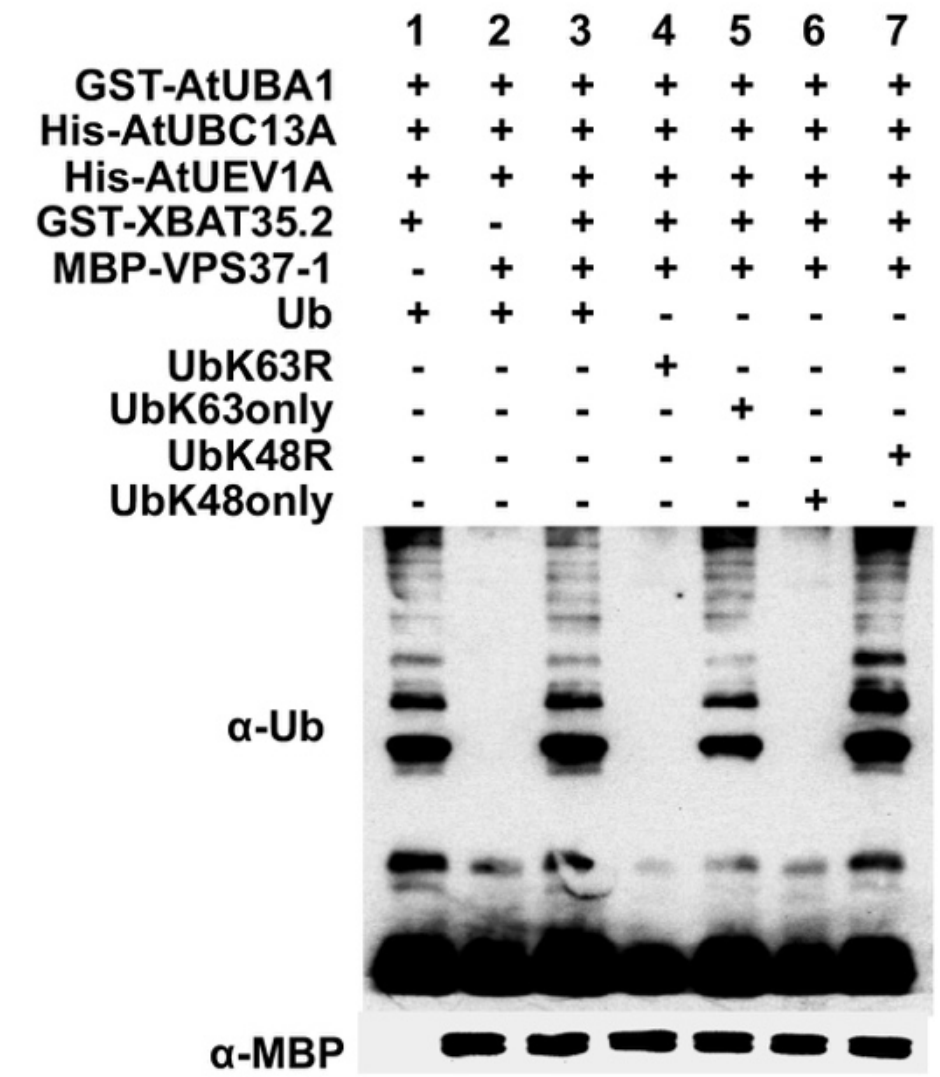

Figure S4. XBAT35.2 does not ubiquitinate VPS37-1 with K63-linked ubiquitin chains.

In vitro ubiquitination of MBP-VPS37-1 by XBAT35.2 was assayed in the presence of an Arabidopsis E1 (AtUBA1), E2 (AtUBC13a, specific for K63-linked ubiquitin chains), E2 variant AtUEV1a. Upper panel: XBAT35.2 autoubiquitination detected by anti-ubiquitin antibody. Lower panel: anti-MBP antibody was used to detect the ubiquitination of MBP-VPS37-1.

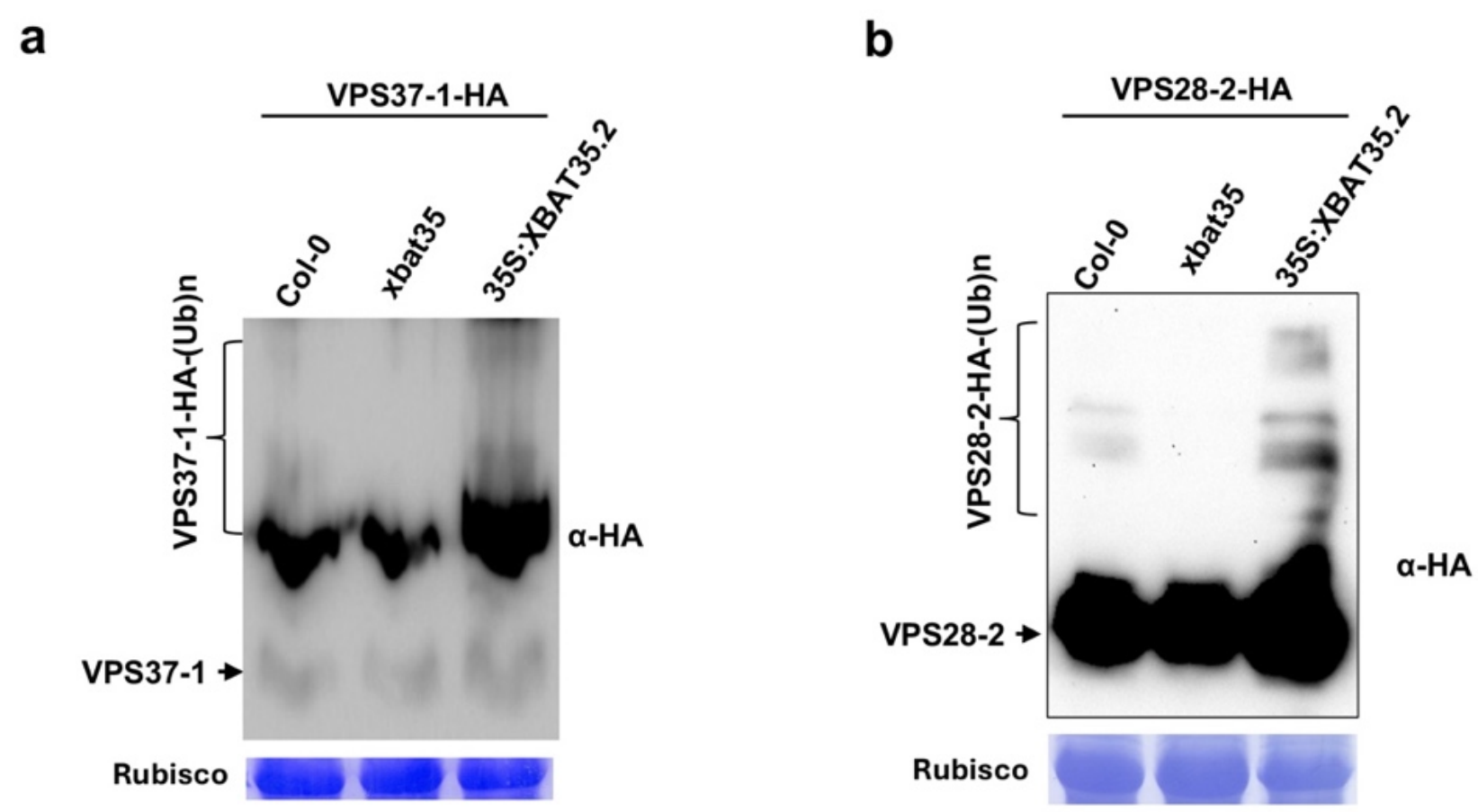

Figure S5. XBAT35.2 directs ubiquitination of VPS28-2 and VPS37-1 *in vivo*.

The ubiquitination of VPS37-1 (**a**) and VPS28-2 (**b**) *in vivo* were detected in seedlings of Arabidopsis transgenic lines where VPS28-2 or VPS37-1-HA was expressed in the genetic background of Col-0, *xbat35*, and 35S:XBAT35.2-FLAG, respectively. The seedlings were grown on ½ MS medium plus agar plates for 10 days under long day condition, then transferred to a flask with liquid ½ MS medium containing 75 μM MG132 and shaken overnight before total proteins extraction and immunoblotting.

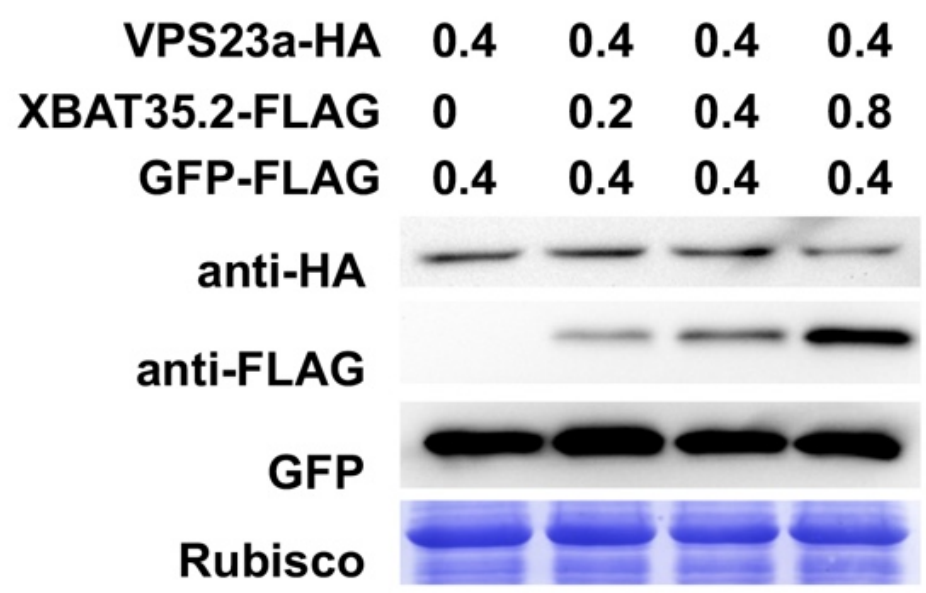

Figure S6. **XBAT35.2 promotes degradation of VPS23a**.

A. tumefaciens cells carrying the 35S:VPS23a-HA construct were co-infiltrated into tobacco leaves with increasing amount of *A. tumefaciens* cells that express XBAT35.2-FLAG. GFP-FLAG was used as internal expression control. Samples were collected 48 h after infiltration, followed by immunoblotting analysis using anti-HA antibodies.

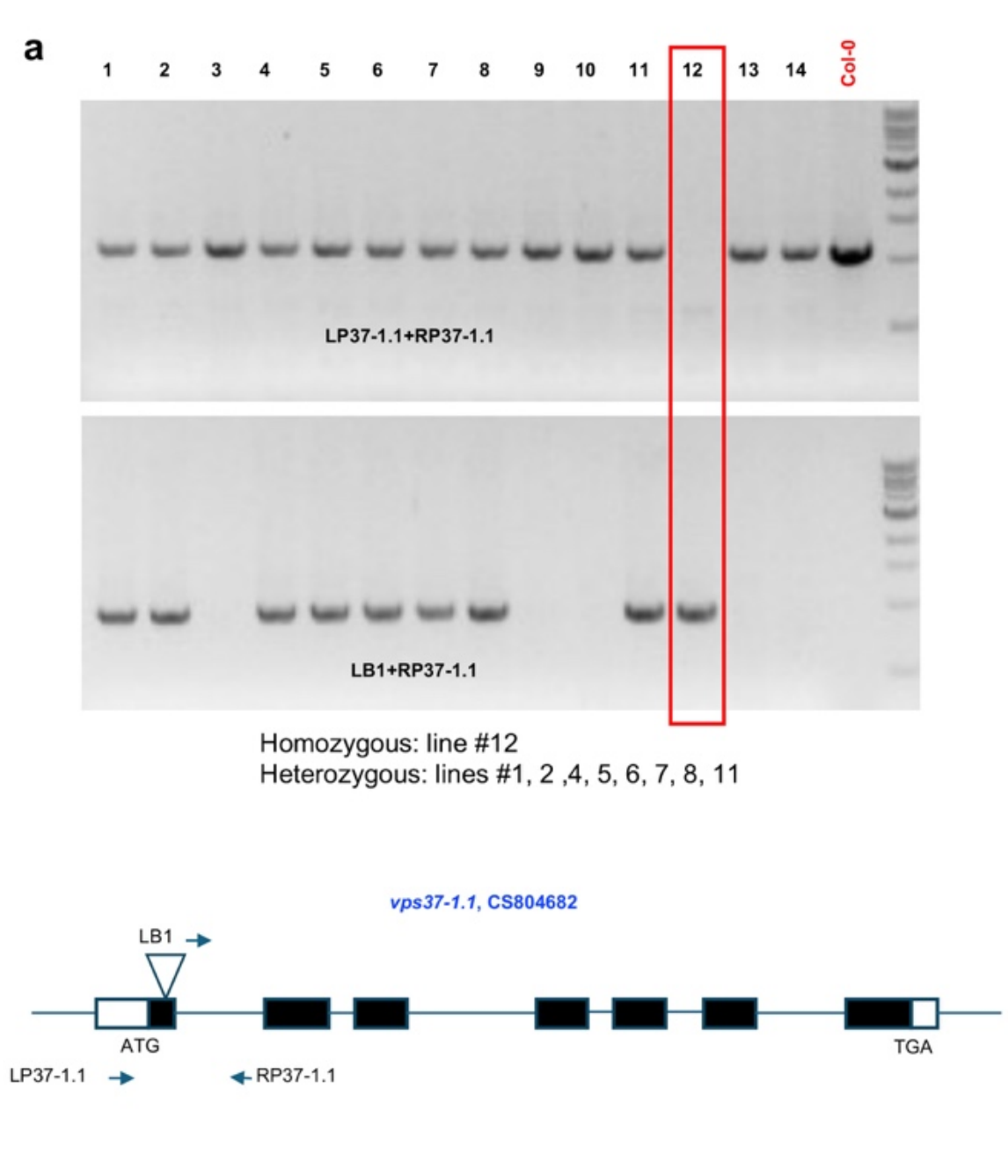

Figure S7. Identification of the Arabidopsis *vps28-2* and *vps37-1.1* T-DNA insertion mutants.

(**a**) Identification of the *vps37-1.1* T-DNA insertion mutant. Upper panel: genotyping the T-DNA insertion using primers indicated in the lower panel. Lower panel: schematic representation of the genomic structure of *VPS37-1* gene with the position of the T-DNA insertion being shown. The gene-specific primers and a primer at the right border of the T-DNA for genotyping are indicated as filled arrows. Filled boxes: exons; open boxes: 5’ and 3’ UTR region.

(**b**) Identification of the *vps28-2* T-DNA insertion mutant. Upper panel: genotyping the T-DNA insertion using primers indicated in the lower panel. Lower panel: schematic representation of the genomic structure of *VPS28-2* gene with the position of the T-DNA insertion being shown. The gene-specific primers and a primer at the right border of the T-DNA for genotyping are indicated as filled arrows. Filled boxes: exon; open boxes: 5’ and 3’ UTR region.

(**c**) Growth phenotype of *Col-0*, the *vps28-2* and *vps37-1.1* single and double (*vps28-2*/*vps37-1.1*) mutants, and *VPS28-2* and *VPS37-1*-overexpressing transgenic line plants. Photo was taken when the plants were approximately 5 weeks old.

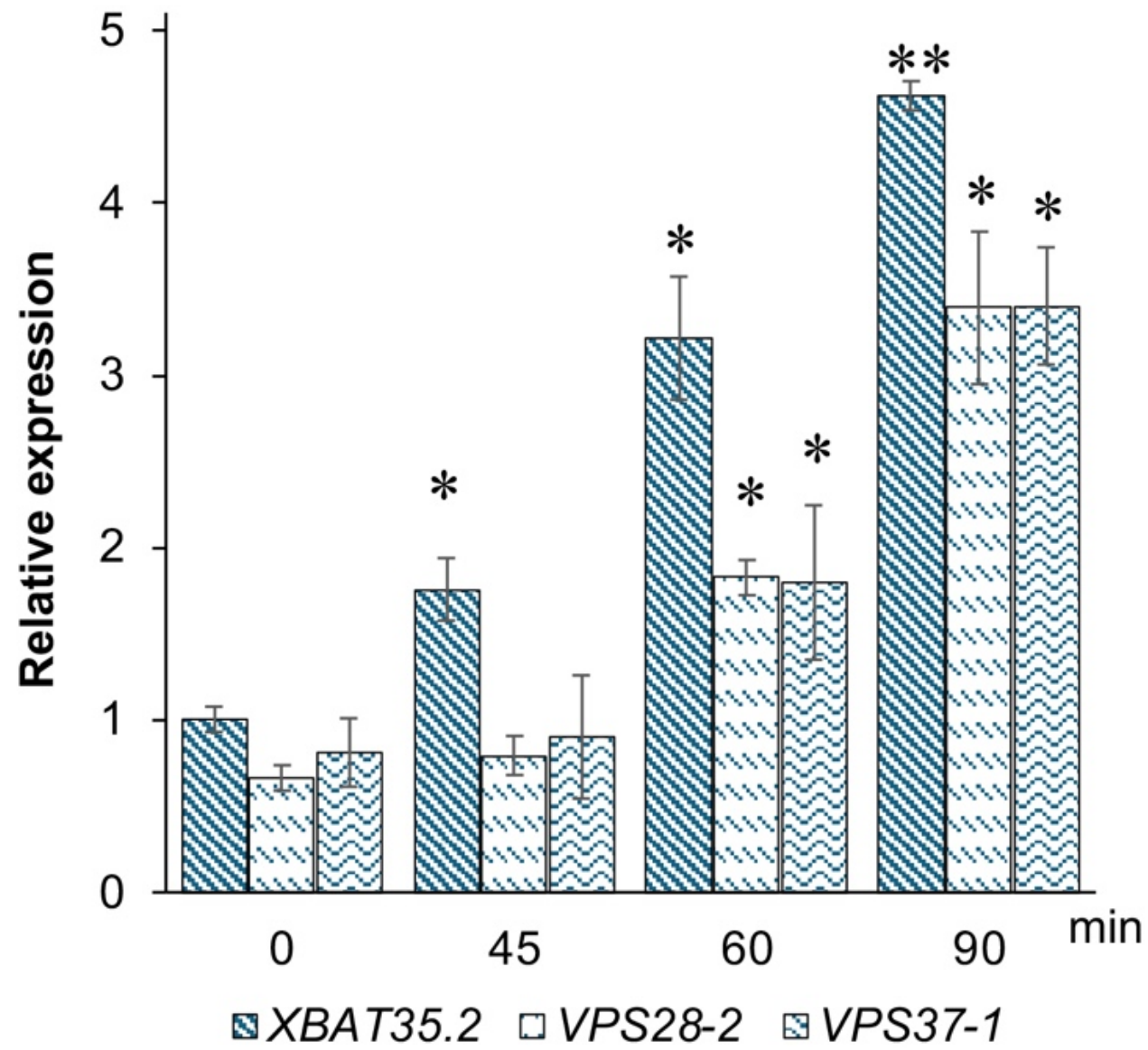

Figure S8. Expression of *XBAT35.2*, *VPS28-2* and *VPS37-1* is induced following *Pst* DC3000 treatment.

Quantitative RT-PCR analysis of *XBAT35.2*, *VPS28-2* and *VPS37-1* expression in *Arabidopsis* Col-0 leaves inoculated with *Pst* DC3000. Error bars represent the SD of 3 replicates. *: P< 0.05, **: P< 0.01 (one-way ANOVA followed by Tukey's HSD test).

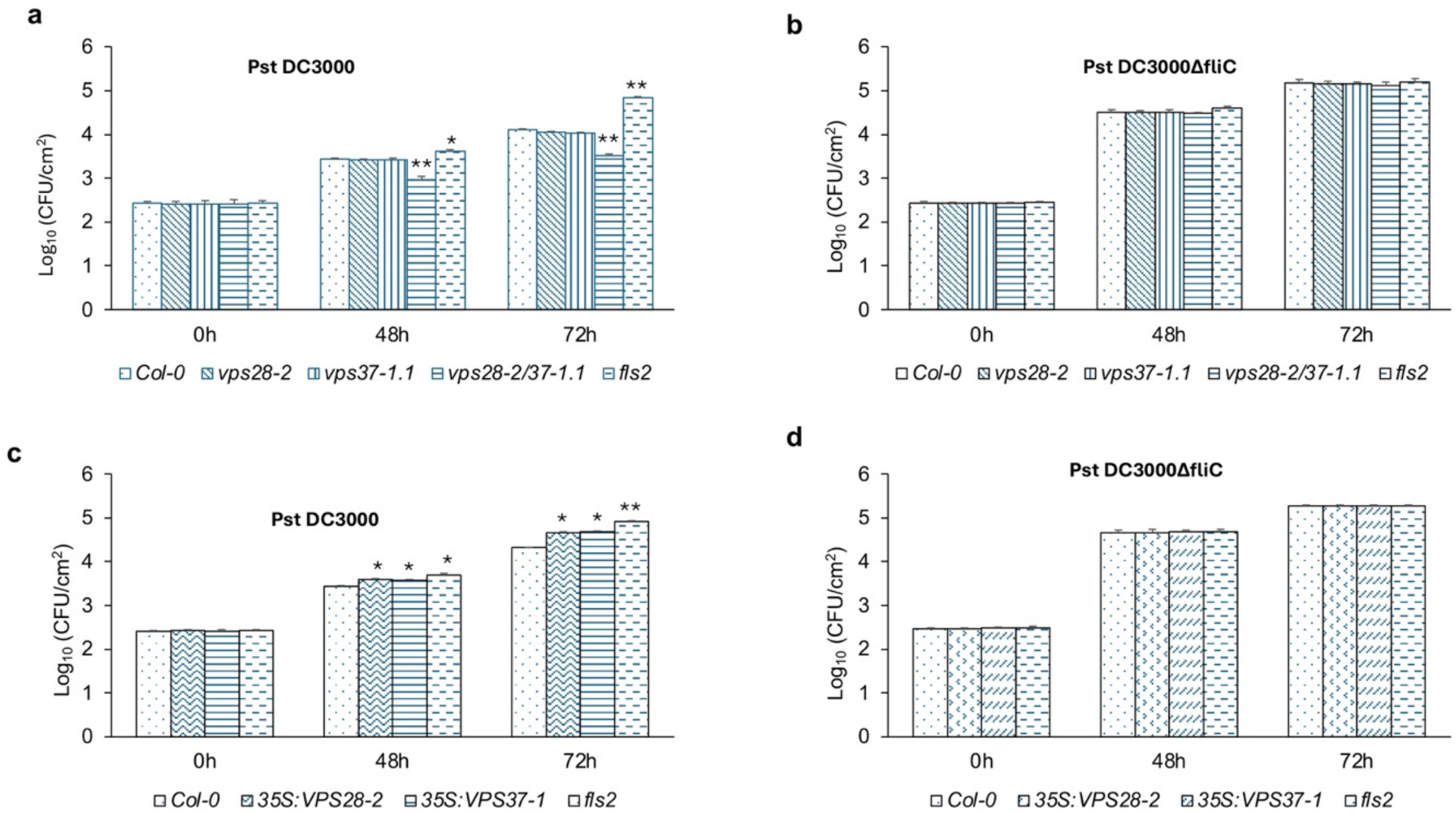

Figure S9. Bacterial pathogen growth in *VPS28-2* and *VPS37-1* loss-of-function and overexpression lines. VPS28-2 and VPS37-1 redundantly modulate plant immunity as shown by the bacterial pathogen growth assay on *Arabidopsis* plants in which *VPS28-2* and *VPS37-1* are loss-of-function (**a** and **b**) or overexpressed (**c** and **d**). The *fls2* mutant was included as control. For this pathogen growth assay, 4-week-old plants were inoculated with *Pst* DC3000 or *Pst* DC3000ΔfliC (OD_{600}=0.2), and the bacterial growth was determined at 48 and 72 hpi. Error bars represent the SD of 9 independent samples for each genotype. *: P< 0.05; **: P< 0.01 (one-way ANOVA followed by Tukey's HSD test).

Dataset S1

A. Arabidopsis and N. benthamiana VPS28-2 Protein Sequence Alignment Using CLUSTAL Omega (version 1.2.4) Algorithm

```
AtVPS28-2(AT4G05000)          ------------------------------------------------------------    0
NbVPS28-2.1(Nbe03g32210)      MSQAKSTDLNFSLLFFVDRLPAATLNFPLFRAACVFHLLLYLTKSSIFARVPSSSPTLLT    60
NbVPS28-2.4(Nbe18g25060)      ------------------------------------------------------------    0
NbVPS28-2.2(Nbe10g10130)      ------------------------------------------------------------    0
NbVPS28-2.3(Nbe17g20060)      ------------------------------------------------------------    0


AtVPS28-2(AT4G05000)          --------MMEVKLWNDKREREMYENFAELFAIIKATEKLEKAYIRDLINPSEYESECQK    52
NbVPS28-2.1(Nbe03g32210)      KVKIVSPTLMEVKLWNDKREREMYDNFAELFAIIKATEKLEKAYVRDIISPAEYETECQK    120
NbVPS28-2.4(Nbe18g25060)      ---------MEVKLWNDKREREMYDNFAELFAIIKATEKLEKAYVRDIISPAEYETECQK    51
NbVPS28-2.2(Nbe10g10130)      --------MMEVKLWNDKREREMYDNFAELFAIIKATEKLEKAYVRDIVSPAVYETECQK    52
NbVPS28-2.3(Nbe17g20060)      ---------MEVKLWNDKREREMYDNFGELFAIIKAAEKLEKAYVRDIVSPAIYETECQK    51
                                       ***************:**.********:*******:**::.*: **:****

AtVPS28-2(AT4G05000)          LIVHFKTLSATLKDTVPNIERFADTYKMDCPAALYRLVTSGLPATVEHRATVAASTSNSA    112
NbVPS28-2.1(Nbe03g32210)      LIAHFKTLSSTLKDTVPSIERFHDTYKMDCPAALNRLVTSGVPATVEHRAATAMSATTSA    180
NbVPS28-2.4(Nbe18g25060)      LIAHFKTLSSTLKDTVPSIERFHDTYKMDCPAALNRLVTSGVPATVEHRAAAAISATTSA    111
NbVPS28-2.2(Nbe10g10130)      LIAQFKTLSSTLKDAVPNIERFHDTYKMDCPAALNRLVTSGVPATVEHRAAASMSAVTSA    112
NbVPS28-2.3(Nbe17g20060)      LIAQFKTLSSTLKDAVPNIERFHDTYKMDCPAALNRLVTSGVPATVEHRAAASMSAITSA    111
                              **.:*****:****:**.**** *********** ******:********:.: *: .**

AtVPS28-2(AT4G05000)          SIVAECVQNFITSMDSLKLNMVAVDQVYPLLSDLSASLNKLSILPPDFEGKTKMKEWLSR    172
NbVPS28-2.1(Nbe03g32210)      AVVAECVQNFITAMDSLKLNMVAVDQVHPLLSDLSSSLNKLSILPADFEGKTKMREWLSR    240
NbVPS28-2.4(Nbe18g25060)      AVVAECVQNFITAMDSLKLNMVAVDQVHPLLSDLSSSLNKLSILPADFEGKTKMREWLSR    171
NbVPS28-2.2(Nbe10g10130)      AVVAECVQNFITAMDSLKLNMVAVDQVHPLLSELSSSLNKLSILPVDFEGKTKMREWLSR    172
NbVPS28-2.3(Nbe17g20060)      AVVAECVQNFITAMDSLKLNMVAVDQVHPLLSELSSSLNKLSILPVDFEGKTKMREWLSR    171
                              ::**********:**************:****:**:********* ********:*****

AtVPS28-2(AT4G05000)          LSKMGAADELTEQQSRQLHFDLESSYNSFMAALPKAGN    210
NbVPS28-2.1(Nbe03g32210)      LSKMGAADELTEQQARQLHFDLESSYNSFMAALPTAGT    278
NbVPS28-2.4(Nbe18g25060)      LSKMGAADELTEQQARQLHFDLESSYNSFMAALPAAGT    209
NbVPS28-2.2(Nbe10g10130)      LSKMGAADELTEQQARQLHFDLESSYNSFMAGLPTDGT    210
NbVPS28-2.3(Nbe17g20060)      LSKMGAADELTEQQARQLHFDLESSYNSFMAALPTDGT    209
                              **************:****************.**  *.
```

The protein sequence alignments were conducted using the Clustal Omega algorithm (Sievers *et al.*, 2011) with default parameters of the software.

B. Percent Identity Matrix of AtVPS28-2 (AT4G05000), NbVPS28-2.1 (Nbe03g32210), NbVPS28-2.2 (Nbe10g10130), NbVPS28- 2.3 (Nbe17g20060) and NbVPS28-2.4 (Nbe18g25060) Protein Sequence Alignment

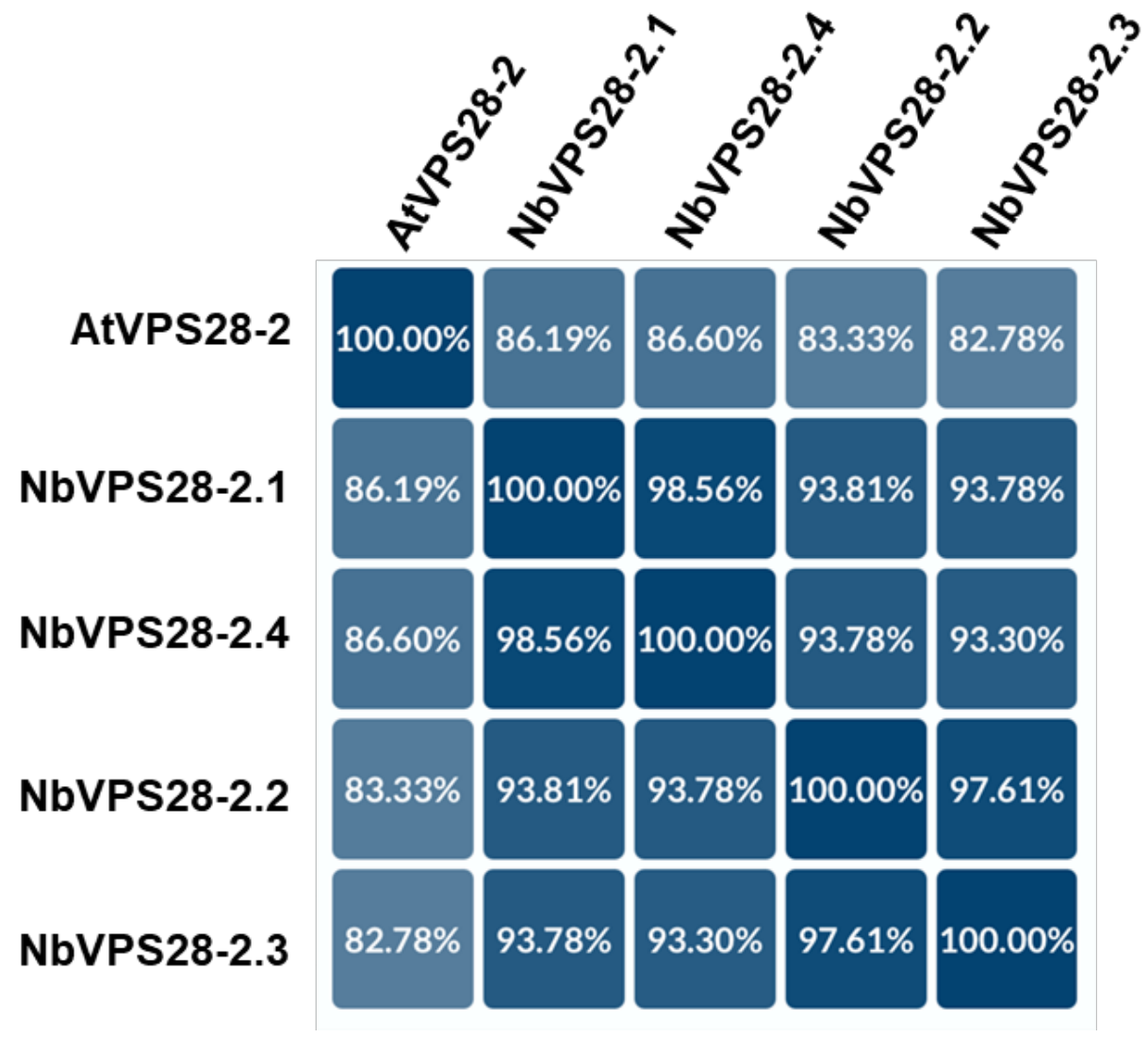

The Percent Identity Matrix of the protein sequences aligned in (**A**). The matrix is calculated by comparing each pair of aligned sequences, counting the number of identical amino acids at corresponding positions, dividing that number by the total length of the alignment, and then multiplying by 100 to get the percentage of identity between the two sequences. The image was generated by the online algorithm available at https://www.uniprot.org/align/.

Reference

Sievers F, Wilm A, Dineen D, Gibson TJ, Karplus K, Li W, Lopez R, McWilliam H, Remmert M, Söding J, et al. 2011. Fast, scalable generation of high-quality protein multiple sequence alignments using Clustal Omega. *Molecular Systems Biology* **7**: 539.

Dataset S2

A. Arabidopsis and N. benthamiana VPS37-1 Protein Sequence Alignment Using CLUSTAL Omega (version 1.2.4) Algorithm

```
AtVPS37-1(AT3G53120)          -MFNFWGSKDQQQGQSRPQEASSQSPWYSPSLVSSPSSSRPQSSG---------------      44
NbVPS37-1.2(Nbe13g03380)      -MFRFWGSEEQQAQ-PNSQE-ANNGSMYSPSVASSPSSSRPATPSSSSSGSFNVQRPTNR      57
NbVPS37-1.1(Nbe10g05990)      MFKQFWGSQGQQAQ-QRPRE-GNNDPWYAPSASSSPGSSRPTTPSPNTSGSFGTQRPTDR      58
NbVPS37-1.3(Nbe15g09500)      MFKQFWGSQGQQAQ-QRPQE-GNNDPWYAPSASISPGSSRPTTPSPSSSGSFGAQRPTDR      58
                               : .****: **    . :* ..:.  *:**   **.**** : .

AtVPS37-1(AT3G53120)          -QISAQVSPGEAAGIIVFLKDKSVDELRKLLSDKDAYQQFLLSLDQVKVQNNIKDELRRE      103
NbVPS37-1.2(Nbe13g03380)      PSSVSHVSPEEAAGTIAALKDKSVDELRKLLSDDAAYRNFLLSLEAVKTQSNVKDELRNE      117
NbVPS37-1.1(Nbe10g05990)      PSSASQVSPAEAACIINALKDKSVDELRKLLSGDDACQNFLLSLEPVKTQNKVRDELRNE      118
NbVPS37-1.3(Nbe15g09500)      PSSASHVSPAEAACIINALKDKSIDELRKLLSGDDAYQNFLLSLEPVKTQNKVRDELRNE      118
                               .  ::*** ***  *  *****:********.. * ::*****: **.*.:::****.*

AtVPS37-1(AT3G53120)          TLQLARDNLEKEPQIMELRNQCRIIRTTELATAQEKLNELERQKEEILKFYSPGSLLHKL      163
NbVPS37-1.2(Nbe13g03380)      TLQLARENLEKEPRIMELRNQCRIIRTTELAVAQEKLHELERRKEELLKLYSPLSLLHHL      177
NbVPS37-1.1(Nbe10g05990)      TLQLARENLEKEPRIMELRNQCRIIRTTELAAAQEKLHELERRKEVLLKLYSPASHLHRL      178
NbVPS37-1.3(Nbe15g09500)      TLQLARENLEKEPRIMELRNQCRIIRTTELAAAQEKLHELERRKEELLKLYSPASLLHRL      178
                              ******:******:*****************.*****:****:** :**:*** * **:*

AtVPS37-1(AT3G53120)          QEAMNQVDEESEALQEKFLEKEIDTAAFVQKYKKLRTTYHRRALIHLAAKTSNI-      217
NbVPS37-1.2(Nbe13g03380)      QDAMKKTEEESEAMLGQLLDREIDLTTFVQKYKKLGCSYHRCSLIHLAAKSSFAA      232
NbVPS37-1.1(Nbe10g05990)      QDAMNKTDEESESIHRQFLDGEIDLTTFVQKYKKLRYSYHKRALTHLAAKTSVTG      233
NbVPS37-1.3(Nbe15g09500)      QDAMNKTDEESESLRRQFLDGEIDLTTFMQKYKKLRYSYHKRALTHLAAKTCVTG      233
                              *:**::.:****::  ::*: *** ::*:******  :**: :* *****:.
```

The protein sequence alignments were conducted using the Clustal Omega algorithm (Sievers *et al.*, 2011) with default parameters of the software.

B. Percent Identity Matrix of AtVPS37-1 (AT3G53120), NbVPS37-1.1(Nbe10g05990), NbVPS37-1.2(Nbe13g03380) and NbVPS37-1.3(Nbe15g09500) Protein Sequence Alignment

	AtVPS37-1	NbVPS37-1.2	NbVPS37-1.1	NbVPS37-1.3
AtVPS37-1	100.00%	66.98%	67.44%	67.44%
NbVPS37-1.2	66.98%	100.00%	73.71%	75.00%
NbVPS37-1.1	67.44%	73.71%	100.00%	93.99%
NbVPS37-1.3	67.44%	75.00%	93.99%	100.00%

The Percent Identity Matrix of the protein sequences aligned in (A). The matrix is calculated by comparing each pair of aligned sequences, counting the number of identical amino acids at corresponding positions, dividing that number by the total length of the alignment, and then multiplying by 100 to get the percentage of identity between the two sequences. The image was generated by the online algorithm available at https://www.uniprot.org/align/.

Reference

Sievers F, Wilm A, Dineen D, Gibson TJ, Karplus K, Li W, Lopez R, McWilliam H, Remmert M, Söding J, et al. 2011. Fast, scalable generation of high-quality protein multiple sequence alignments using Clustal Omega. *Molecular Systems Biology* **7**: 539.

Dataset S3

A. Visualization and validation of NbVPS37-1, and VPS23a sequences using Scaffold 5 software.

Peptides highlighted in yellow indicate sequences identified by MS/MS.

NbVPS37-1

Nbe10g05990.1 (100%), 26,434.4 Da
gene=Nbe10g05990
3 exclusive unique peptides, 4 exclusive unique spectra, 4 total spectra, 9/233 amino acids (4% coverage)

```
MFKQFWGSQG QQAQQRPREG NNDPWYAPSA SSSPGSSRPT TPSPNTSGSF GTQRPTDRPS
SASQVSPAEA ACIINALKDK SVDELRKLLS GDDACQNFLL SLEPVKTQNK VRDELRNETL
QLARENLEKE PRIMELRNQC RIIRTTELAA AQEKLHELER RKEVLLKLYS PASHLHRLQD
AMNKTDEESE SIHRQFLDGE IDLTTFVQKY KKLRYSYHKR ALTHLAAKTS VTG*
```

NbVPS23a.1

Nbe13g03040.1 (100%), 45,510.2 Da
gene=Nbe13g03040
7 exclusive unique peptides, 18 exclusive unique spectra, 49 total spectra, 250/404 amino acids (62% coverage)

```
MMPPPNSAQY TQQFLSSVLS QRGPSALPYT EDVKWLIRQH LLSLIETYPS LQPKTATFTH
NDGRTVNLLQ ADGTVPMVYQ DVTYNIPVII WLMESYPRHS PLVFVNPTHD MIIKRPHQFV
NPSGVVSIPY LQNWIYPSSN LVELARNLSH FFGRDPPLYA QRRPNPNPSP SPNPHPPPRT
SFSNNNSPVG PGGVIRPAIP PRAFPQSPYG SGSTGRIMDD PSEVYKRNAI DKLVVKLHSD
ILGLREAREA DMEGLFNAQG VLRQREERLD EGLKEMQYEK EGLEQQLQMV LMNSDVLEGW
LRENEAKMTN LGNVDVDTAF EPCDHLSKQM LDCTASDLAI DDVIYSLDKA IQDGTIPFDQ
YLRNVRSLSR EQFFHRATAS KVRAAQMQAQ VANMASRASS PYAL*
```

NbVPS23a.2

Nbe13g03040.1 (100%), 45,510.2 Da
gene=Nbe13g03040
7 exclusive unique peptides, 18 exclusive unique spectra, 49 total spectra, 250/404 amino acids (62% coverage)

```
MMPPPNSAQY TQQFLSSVLS QRGPSALPYT EDVKWLIRQH LLSLIETYPS LQPKTATFTH
NDGRTVNLLQ ADGTVPMVYQ DVTYNIPVII WLMESYPRHS PLVFVNPTHD MIIKRPHQFV
NPSGVVSIPY LQNWIYPSSN LVELARNLSH FFGRDPPLYA QRRPNPNPSP SPNPHPPPRT
SFSNNNSPVG PGGVIRPAIP PRAFPQSPYG SGSTGRIMDD PSEVYKRNAI DKLVVKLHSD
ILGLREAREA DMEGLFNAQG VLRQREERLD EGLKEMQYEK EGLEQQLQMV LMNSDVLEGW
LRENEAKMTN LGNVDVDTAF EPCDHLSKQM LDCTASDLAI DDVIYSLDKA IQDGTIPFDQ
YLRNVRSLSR EQFFHRATAS KVRAAQMQAQ VANMASRASS PYAL*
```

B. Visualization and validation of NbVPS37-1, NbVPS28-2, and VPS23a sequences using Scaffold 5 software.

Peptides highlighted in yellow indicate sequences identified by MS/MS.

NbVPS37-1

Nbe10g05990.1 gene=Nbe10g05990 (100%), 26,434.4 Da
Nbe10g05990.1 gene=Nbe10g05990
2 exclusive unique peptides, 2 exclusive unique spectra, 2 total spectra, 9/233 amino acids (4% coverage)

```
MFKQFWGSQG QQAQQRPREG NNDPWYAPSA SSSPGSSRPT TPSPNTSGSF GTQRPTDRPS
SASQVSPAEA ACIINALKDK SVDELRKLLS GDDACQNFLL SLEPVKTQNK VRDELRNETL
QLARENLEKE PRIMELRNQC RIIRTTELAA AQEKLHELER RKEVLLKLYS PASHLHRLQD
AMNKTDEESE SIHRQFLDGE IDLTTFVQKY KKLRYSYHKR ALTHLAAKTS VTG*
```

NbVPS28-2

Nbe10g10130.1 gene=Nbe10g10130 (100%), 23,489.3 Da
Nbe10g10130.1 gene=Nbe10g10130
2 exclusive unique peptides, 2 exclusive unique spectra, 2 total spectra, 20/210 amino acids (10% coverage)

```
MMEVKLWNDK REREMYDNFA ELFAIIKATE KLEKAYVRDI VSPAVYETEC QKLIAQFKTL
SSTLKDAVPN IERFHDTYKM DCPAALNRLV TSGVPATVEH RAAASMSAVT SAAVVAECVQ
NFITAMDSLK LNMVAVDQVH PLLSELSSSL NKLSILPVDF EGKTKMREWL SRLSKMGAAD
ELTEQQARQL HFDLESSYNS FMAGLPTDGT *
```

NbVPS23a.1

Nbe13g03040.1 gene=Nbe13g03040 (100%), 45,510.2 Da
Nbe13g03040.1 gene=Nbe13g03040
2 exclusive unique peptides, 2 exclusive unique spectra, 12 total spectra, 116/404 amino acids (29% coverage)

```
MMPPPNSAQY TQQFLSSVLS QRGPSALPYT EDVKWLIRQH LLSLIETYPS LQPKTATFTH
NDGRTVNLLQ ADGTVPMVYQ DVTYNIPVII WLMESYPRHS PLVFVNPTHD MIIKRPHQFV
NPSGVVSIPY LQNWIYPSSN LVELARNLSH FFGRDPPLYA QRRPNPNPSP SPNPHPPPRT
SFSNNNSPVG PGGVIRPAIP PRAFPQSPYG SGSTGRIMDD PSEVYKRNAI DKLVVKLHSD
ILGLREAREA DMEGLFNAQG VLRQREERLD EGLKEMQYEK EGLEQQLQMV LMNSDVLEGW
LRENEAKMTN LGNVDVDTAF EPCDHLSKQM LDCTASDLAI DDVIYSLDKA IQDGTIPFDQ
YLRNVRSLSR EQFFHRATAS KVRAAQMQAQ VANMASRASS PYAL*
```

NbVPS23a.2

Nbe13g03040.1 gene=Nbe13g03040 (100%), 45,510.2 Da
Nbe13g03040.1 gene=Nbe13g03040
2 exclusive unique peptides, 2 exclusive unique spectra, 12 total spectra, 116/404 amino acids (29% coverage)

```
MMPPPNSAQY TQQFLSSVLS QRGPSALPYT EDVKWLIRQH LLSLIETYPS LQPKTATFTH
NDGRTVNLLQ ADGTVPMVYQ DVTYNIPVII WLMESYPRHS PLVFVNPTHD MIIKRPHQFV
NPSGVVSIPY LQNWIYPSSN LVELARNLSH FFGRDPPLYA QRRPNPNPSP SPNPHPPPRT
SFSNNNSPVG PGGVIRPAIP PRAFPQSPYG SGSTGRIMDD PSEVYKRNAI DKLVVKLHSD
ILGLREAREA DMEGLFNAQG VLRQREERLD EGLKEMQYEK EGLEQQLQMV LMNSDVLEGW
LRENEAKMTN LGNVDVDTAF EPCDHLSKQM LDCTASDLAI DDVIYSLDKA IQDGTIPFDQ
YLRNVRSLSR EQFFHRATAS KVRAAQMQAQ VANMASRASS PYAL*
```

Dataset S4

A. Visualization and validation of XBAT35.2 (AT3G23280.2) sequences using Scaffold 5 software.

Peptides highlighted in yellow indicate sequences identified by MS/MS.

AT3G23280.2 (100%), 47,142.9 Da
| Symbols:XBAT35 | XB3 ortholog 5 in Arabidopsis thaliana | Chr3:8321588-8324109 FORWARD LENGTH=438
27 exclusive unique peptides, 66 exclusive unique spectra, 135 total spectra, 272/438 amino acids (62% coverage)

```
MGQQQSKGEL LYQQVSYGNS EGIRALHRDG GDLEWMDREG KTPLILACMN SELFDVAKTL
IELGSNVNAY RPGRHAGTPL HHAAKRGLEN TVKLLLSHGA NPLVLNDDCQ TPLEVARVKG
FSNVVRAIEK HICLFSGWMR EFYGPTFLDL FAPQLLSRRV WVVIVPTGSR NPTKPFKLEL
VVYASLQDAQ PRTVMPLWKA NLEEPKAKQS DTSVMIVDNS TKTRLKFAPS TEGDSQQLKW
FCDACKGIPQ PMHPPVFLQA PPSAPPPPSE DGLAMGMNAS LHTTMSDPSN LNHHSIGQAS
SSSGPSSSTA PPSGKASAFG FNSHGIGIVL ESSPSAPPLT DDDIATVDDG PIHYPSIDST
PVDLPSAASL PASTEGERKE DGNTGTCAIC LDAPSEAVCV PCGHVAGCMS CLKEIKSKNW
GCPVCRAKID QVIKLYRV
```

B. Visualization and validation of VPS28-2 (AT4G05000) sequences using Scaffold 5 software.

Peptides highlighted in yellow indicate sequences identified by MS/MS.

AT4G05000.1 (100%), 23,690.1 Da
| Symbols:VPS28-2,VPS28-1 | vacuolar protein sorting-associated protein 28 homolog 1 | Chr4:2563953-2564585 FORWARD LENGTH=210
12 exclusive unique peptides, 24 exclusive unique spectra, 49 total spectra, 168/210 amino acids (80% coverage)

```
MMEVKLWNDK REREMYENFA ELFAIIKATE KLEKAYIRDL INPSEYESEC QKLIVHFKTL
SATLKDTVPN IERFADTYKM DCPAALYRLV TSGLPATVEH RATVAASTSN SASIVAECVQ
NFITSMDSLK LNMVAVDQVY PLLSDLSASL NKLSILPPDF EGKTKMKEWL SRLSKMGAAD
ELTEQQSRQL HFDLESSYNS FMAALPKAGN
```

C. Visualization and validation of VPS37-1 (AT3G53120) sequences using Scaffold 5 software.

Peptides highlighted in yellow indicate sequences identified by MS/MS.

AT3G53120.1 (100%), 24,940.9 Da
| Symbols:VPS37-1 | | Chr3:19690729-19692490 REVERSE LENGTH=217
17 exclusive unique peptides, 31 exclusive unique spectra, 31 total spectra, 175/217 amino acids (81% coverage)

```
MFNFWGSKDQ QQGQSRPQEA SSQSPWYSPS LVSSPSSSRP QSSGQISAQV SPGEAAGIIV
FLKDKSVDEL RKLLSDKDAY QQFLLSLDQV KVQNNIKDEL RRETLQLARD NLEKEPQIME
LRNQCRIIRT TELATAQEKL NELERQKEEI LKFYSPGSLL HKLQEAMNQV DEESEALQEK
FLEKEIDTAA FVQKYKKLRT TYHRRALIHL AAKTSNI
```

Table S1. Primers used for genotyping, cloning, qRT-PCR, and other experiments for this study.

Gene name	Primers
**Primers for genotyping mutant lines**	
LB1	GCCTTTTCAGAAATGGATAAATAGCCTTGCTTCC
LBb1.3	ATTTTGCCGATTTCGGAAC
vps28-2_LP	TCAAATTAATAAAATTTCACGGTCC
vps28-2_RP	GACAAACGCGAAAGAGAGATG
vps37-1.1_LP	TGGAGGATCTGATGGAGAATG
vps37-1.1_RP	GAGCAGAAATTGCTGATACGC
**Primers for molecular cloning**	
AtUBA1-GST-F	CACCATGCTTCACAAGCGAGCTAGTGAAGC
AtUBA1-GST-R	TCACCTGAAGTAGATAGAGACGAG
UBC12-HIS-F	CACCATGGCTTCTAAGAGGATTTCAA
UBC12-HIS-R	TCATCCCATCGCGTACTTCTGTGT
UBC13A-HIS-F	CACCATGGCCAACAGTAATTTGCCG
UBC13A-HIS-R	TCATGCGCCGCTTGCATAAAGACG
UEV1A-HIS-F	CACCATGAGTTCGGAGGAAGCCAAG
UEV1A-HIS-R	TCACATCACACAACATTTAGCTG
XBAT35.2-GST-F	CACCATGGGACAACAGCAATCAAAAGGGGAA
XBAT35.2-GST-R	TCAGACACGGTACAGCTTAATAACCTGA
XBAT35.2-BiFC-F	CACCTCTAGAATGGGACAACAGCAATCAAAAGGGG
XBAT35.2-BiFC-R	GGTACCGACACGGTACAGCTTAATAACCTG
FLS2-BiFC-F	CACCACTAGTATGAAGTTACTCTCAAAGACCTTTTTGATA
FLS2-BiFC-R	GGTACCAACTTCTCGATCCTCGTTACGATCTTCTCG
GFP-TEX/FLAG-F	CACCGGTACCATGGTGAGCAAGGGCGA
GFP-TEX/FLAG-R	CCTTCTAGACTTGTACAGCTCGTCCATGC
XBAT35.2-TEX/FLAG-F	CACCTCTAGAGGTACCATGGGACAACAGCAATCAA
XBAT35.2-TEX/FLAG-R	CCTTCTAGAGACACGGTACAGCTTAATAACCT
XBAT35.2ΔANK-BiFC-F	CACCTCTAGAATGGGACAACAGCAATCACTTAATGATGATTGTC AGACA
XBAT35.2ΔANK-BiFC-R	GGTACCGACACGGTACAGCTTAATAACCTGATCGAT
XBAT35.2ΔRING-BiFC-F	CACCTCTAGAATGGGACAACAGCAATCAAAAGGGG
XBAT35.2ΔRING-BiFC-R	GGTACCCCCGTCTTCTTTCCTTTCAC
XBAT35.2ΔANK-BTEX/FLAG-F	CACCGGTACCATGGAGAAACACATCTGCTTGTTCTC
XBAT35.2ΔANK-BTEX/FLAG-R	CCTTCTAGAGACACGGTACAGCTTAATAACCT
XBAT35.2ΔRING-BTEX/FLAG-F	CACCGGTACCGGGCAGCAGCAATCCAAAGGCGAGCTT
XBAT35.2ΔRING-BTEX/FLAG-R	CCTTCTAGACCCGTCTTCTTTCCTTTCAC
VPS28-2-BiFC-F	CACCTCTAGAATGATGGAGGTCAAATTATGGAACGAC
VPS28-2-BiFC-R	GGTACCATTACCAGCTTTAGGCAAAGCTGCCAT

VPS37-1-BiFC-F	CACCTCTAGAATGTTCAATTTCTGGGGATCAAAAGACC
VPS37-1-BiFC-R	GGTACCAATGTTTGACGTTTTAGCGGCAAGATGGAT
DRP1A-BiFC-F	CACCAGGCCTATGGAAAATCTGATCTCTCTGGTTAACAAG
DRP1A-BiFC-R	CTCGAGCTTGGACCAAGCAACAGCATCGATCTCGGA
DRP2B-BiFC-F	CACCGGATCCATGGAGGCGATCGATGAGTTGTCTCAGC
DRP2B-BiFC-R	GGTACCATACCTGTAAGATGATCCAGACTGTGGC
VPS28-2-BTEX/HA-F	GGTACCATGATGGAGGTCAAATTATGGAACGAC
VPS28-2-BTEX/HA-R	ATTACCAGCTTTAGGCAAAGCTGCCAT
VPS28-2-BTEX/FLAG-F	GGTACCATGTTCAATTTCTGGGGATCAAAAGACC
VPS28-2-BTEX/FLAG-R	TCTAGAAATGTTTGACGTTTTAGCGGCAAGATGG
VPS37-1-BTEX/HA-F	GGTACCATGTTCAATTTCTGGGGATCAAAAGACC
VPS37-1-BTEX/HA-R	AATGTTTGACGTTTTAGCGGCAAGATGG
VPS23A-BTEX/HA-F	GGTACCATGGTTCCCCCGCCGTCTAATCCGCAGCA
VPS23A-BTEX/HA-R	TGAATGTAACCTACCTGCGATGGCTGCAAC
XBAT35.2-GFP-F	CACCATGGGACAACAGCAATCAAAAGGGGAA
XBAT35.2-GFP-R	GACACGGTACAGCTTAATAACC
VPS28-2-MBP-F	CACCATGATGGAGGTCAAATTATGGAACGAC
VPS28-2-MBP-R	TTAATTACCAGCTTTAGGCAAAGCTGCC
VPS37-1-MBP-F	CACCTTCAATTTCTGGGGATCAAAAGACC
VPS37-1-MBP-R	TCAAATGTTTGACGTTTTAGCGGCAAGATGG
FLS2CD-BTEX/HA-F	CACCGGTACCATGAAAAAAGAAAAAAAGATTGAAAATTC
FLS2CD-BTEX/HA-R	CTAAACTTCTCGATCCTCGTTACGATC
**Primers for site-directed mutagenesis**	
XBAT35.2^{AA}-BTEX/FLAG_F	CACCGGTACCATGGGACAACAGCAATCAAAAGGGGAATT
XBAT35.2^{AA}-BTEX/FLAG_R	TCTAGAGACACGGTACAGCTTAATAACCTGATCGA
XBAT35.2^{AA}-GFP-F	CACCGGTACCATGGGACAACAGCAATCAAAAGGGGAATT
XBAT35.2^{AA}-GFP-R	TCTAGAGACACGGTACAGCTTAATAACCTGATCGA
**Primers for qRT-PCR and RT-PCR**	
Actin (At3g18780)-qF	TGTGCCAATCTACGAGGGTTT
Actin (At3g18780)-qR	TTTCCCGCTCTGCTGTTGT
XBAT35.2-qF	CTCCACAAAAACACGTCTTA
XBAT35.2-qR	TTCCAGTGTTCCCGTCTT
VPS28-2-qF	AGAGTACGAATCTGAATG
VPS28-2-qR	AGATCAAAGTGAAGTTGC
VPS37-1-qF	AAGCGTGGATGAACTCAG
VPS37-1-qR	GACGTTTTAGCGGCAAGAT
At2g17740_qF	TGCTCCATCTCTCTTTGTGC
At2g17740_qR	ATGCGTTGCTGAAGAAGAGG
FRK1_qF	ATCTTCGCTTGGAGCTTCTC
FRK1_qR	TGCAGCGCAAGGACTAGAG

Table S2. Representative *Nicotiana benthamiana* immunity- and ESCRT-related proteins co-immunoprecipitated with XBAT35.2-FLAG in the Co-IP/LC-MS/MS assays

Protein	*Arabidopsis thaliana* accession	*N. benthamiana* accession	references
**UBP12***	AT5G06600	Nbe16g28690; Nbe06g32030; Nbe13g07220; Nbe10g04880; Nbe19g03900; Nbe09g13970	(Liu *et al.*, 2022; Zhou *et al.*, 2023)
**UBP13***	AT3G11910		
**HIR3**	AT5G51570	Nbe06g14870; Nbe05g17440; Nbe05g26050; Nbe02g27250; Nbe05g21380	(Li *et al.*, 2019; Liu *et al.*, 2024)
**RD19**	AT4G39090	Nbe02g13940; Nbe01g01530; Nbe07g19880; Nbe14g17540	(Bernoux *et al.*, 2008)
**ACD11***	AT2G34690	Nbe03g26890; Nbe04g14220; Nbe10g14130	(Li *et al.*, 2020)
**ADF2**	AT3G46000	Nbe15g30500; Nbe12g15360; Nbe18g10550; Nbe14g06650	(Clement *et al.*, 2009)
**PBL1**	AT3G55450	Nbe19g08510; Nbe07g02630; Nbe08g18720; Nbe14g07270; Nbe18g11100; Nbe12g35000	(Zhang *et al.*, 2010; Song *et al.*, 2026)
**VPS2.2**	AT5G44560	Nbe03g26430; Nbe04g13750; Nbe03g12540; Nbe04g01140; Nbe09g03640	(Ibl *et al.*, 2012)
**VPS4**	AT2G27600	Nbe10g12430; Nbe19g35330; Nbe06g33920; Nbe11g25370	(Buono *et al.*, 2016)
**VPS23A***	AT3G12400	Nbe08g03450; Nbe13g03040; Nbe13g09380; Nbe17g07620	(Lou *et al.*, 2020; Liu *et al.*, 2022)
**VPS28-2****	AT4G05000	Nbe03g32210; Nbe10g10130; Nbe18g25060; Nbe17g20060	(Spallek *et al.*, 2013)
**VPS37-1****	AT3G53120	Nbe10g05990; Nbe15g09500; Nbe13g03380	(Spallek *et al.*, 2013)
**VPS46.2**	AT1G73030	Nbe06g27630; Nbe05g33530; Nbe05g01290	(Zuo *et al.*, 2026)
**ALIX**	AT1G15130	Nbe07g05770; Nbe19g29600; Nbe16g31270; Nbe15g10620	(Cardona-Lopez *et al.*, 2015)

*Proteins showed interaction with XBAT35.2 in prior studies.

**Proteins showed interaction with XBAT35.2 in this study.

Reference

Bernoux M, Timmers T, Jauneau A, Briere C, de Wit PJ, Marco Y, Deslandes L. 2008. RD19, an Arabidopsis cysteine protease required for RRS1-R-mediated resistance, is relocalized to the nucleus by the Ralstonia solanacearum PopP2 effector. *Plant Cell* **20**(8): 2252–2264.

Buono R, Paez-Valencia J, Miller N, Goodman K, Spitzer C, Spalding E, Otegui M. 2016. Role of SKD1 Regulators LIP5 and IST1-LIKE1 in Endosomal Sorting and Plant Development. *Plant Physiology* **171**(1): 251–264.

Cardona-Lopez X, Cuyas L, Marin E, Rajulu C, Irigoyen ML, Gil E, Puga MI, Bligny R, Nussaume L, Geldner N, et al. 2015. ESCRT-III-Associated Protein ALIX Mediates High-Affinity Phosphate Transporter Trafficking to Maintain Phosphate Homeostasis in Arabidopsis. *Plant Cell* **27**(9): 2560–2581.

Clement M, Ketelaar T, Rodiuc N, Banora MY, Smertenko A, Engler G, Abad P, Hussey PJ, de Almeida Engler J. 2009. Actin-depolymerizing factor2-mediated actin dynamics are essential for root-knot nematode infection of Arabidopsis. *Plant Cell* **21**(9): 2963–2979.

Ibl V, Csaszar E, Schlager N, Neubert S, Spitzer C, Hauser MT. 2012. Interactome of the plant-specific ESCRT-III component AtVPS2.2 in Arabidopsis thaliana. *Journal of Proteome Research* **11**(1): 397–411.

Li Q, Serio RJ, Schofield A, Liu H, Rasmussen SR, Hofius D, Stone SL. 2020. Arabidopsis RING-type E3 ubiquitin ligase XBAT35.2 promotes proteasome-dependent degradation of ACD11 to attenuate abiotic stress tolerance. *Plant Journal* **104**(6): 1712–1723.

Li S, Zhao J, Zhai Y, Yuan Q, Zhang H, Wu X, Lu Y, Peng J, Sun Z, Lin L, et al. 2019. The hypersensitive induced reaction 3 (HIR3) gene contributes to plant basal resistance via an EDS1 and salicylic acid-dependent pathway. *Plant Journal* **98**(5): 783–797.

Liu G, Liang J, Lou L, Tian M, Zhang X, Liu L, Zhao Q, Xia R, Wu Y, Xie Q, et al. 2022. The deubiquitinases UBP12 and UBP13 integrate with the E3 ubiquitin ligase XBAT35.2 to modulate VPS23A stability in ABA signaling. *Sci Adv* **8**(14): eabl5765.

Liu X, Zhao H, Yuan M, Li P, Xie J, Fu Y, Li B, Yu X, Chen T, Lin Y, et al. 2024. An effector essential for virulence of necrotrophic fungi targets plant HIRs to inhibit host immunity. *Nat Commun* **15**(1): 9391.

Lou L, Yu F, Tian M, Liu G, Wu Y, Wu Y, Xia R, Pardo JM, Guo Y, Xie Q. 2020. ESCRT-I Component VPS23A Sustains Salt Tolerance by Strengthening the SOS Module in Arabidopsis. *Mol Plant* **13**(8): 1134–1148.

Song B, Choi S, Kong L, Kim SI, Fliegmann J, Li X, Gao Y, DeFalco TA, Hu M, Li M, et al. 2026. New alleles of Arabidopsis BIK1 reinforce its predominant role in pattern-triggered immunity and caution interpretations of other reported functions. *Nat Plants* **12**(2): 284–293.

Spallek T, Beck M, Ben Khaled S, Salomon S, Bourdais G, Schellmann S, Robatzek S. 2013. ESCRT-I mediates FLS2 endosomal sorting and plant immunity. *PLoS Genetics* **9**(12): e1004035.

Zhang J, Li W, Xiang T, Liu Z, Laluk K, Ding X, Zou Y, Gao M, Zhang X, Chen S, et al. 2010. Receptor-like cytoplasmic kinases integrate signaling from multiple plant immune receptors and are targeted by a *Pseudomonas syringae* effector. *Cell Host & Microbe* **7**(4): 290–301.

Zhou Y, Park SH, Chua NH. 2023. UBP12/UBP13-mediated deubiquitination of salicylic acid receptor NPR3 suppresses plant immunity. *Mol Plant* **16**(1): 232–244.

Zuo C, Li S, He Q, Wang T, Wang L, Yang K, Le J. 2026. VPS46, an ESCRT-III-associated regulator, orchestrates ERf-SERKs receptor trafficking to ensure stomatal patterning in Arabidopsis. *New Phytologist* **250**(2): 1111–1127.
